# Effects of Chinese herbal medicines on the occurrence of diabetic retinopathy in type 2 diabetes patients and protection of ARPE-19 retina cells by inhibiting oxidative stress

**DOI:** 10.18632/oncotarget.18846

**Published:** 2017-06-29

**Authors:** Fuu-Jen Tsai, Te-Mao Li, Cheng-Hang Ko, Chi-Fung Cheng, Tsung-Jung Ho, Xiang Liu, Hsinyi Tsang, Ting-Hsu Lin, Chiu-Chu Liao, Ju-Pi Li, Shao-Mei Huang, Jung-Chun Lin, Chih-Chien Lin, Wen-Miin Liang, Ying-Ju Lin

**Affiliations:** ^1^ School of Chinese Medicine, China Medical University, Taichung, Taiwan; ^2^ Genetic Center, Department of Medical Research, China Medical University Hospital, Taichung, Taiwan; ^3^ Department of Health and Nutrition Biotechnology, Asia University, Taichung, Taiwan; ^4^ Department of Chinese Medicine, China Medical University Hospital, Taichung, Taiwan; ^5^ Graduate Institute of Biostatistics, School of Public Health, China Medical University, Taichung, Taiwan; ^6^ National Institute of Allergy and Infectious Diseases, National Institutes of Health, Bethesda, MD, USA; ^7^ Rheumatism Research Center, China Medical University Hospital, Taichung, Taiwan; ^8^ School of Medical Laboratory Science and Biotechnology, College of Medical Science and Technology, Taipei Medical University, Taipei, Taiwan; ^9^ Department of Cosmetic Science, Providence University, Taichung, Taiwan

**Keywords:** type 2 diabetes, diabetic retinopathy, Chinese herbal medicine, oxidative stress, retina cells

## Abstract

Diabetic retinopathy is a microvascular complication of type 2 diabetes and the leading cause of acquired blindness. In Taiwan, Chinese herbal medicine (CHM) is a popular adjunctive therapy. In this study, we investigated the CHM prescription patterns and their effects. We identified 23,701 subjects with type 2 diabetes in a database, and after matching for age and gender, 6,948 patients each were assigned to CHM and non-CHM groups. In the female subgroups, the cumulative retinopathy probability was lower for the CHM users than that for the CHM non-users (*P* < 0.001, log-rank test). Among the top 10 CHMs, Jia-Wei-Xiao-Yao-San (JWXYS; 52.9%), Shu-Jing-Huo-Xue-Tang (SJHXT; 45.1%), and Ge-Gen-Tang (GGT; 43.7%) were the most common herbal formulas. Yan-Hu-Suo (48.1%), Ge-Gen (42.1%), and Huang-Qin (HQin; 40.1%) were the most common single herbs. CHM network analysis showed that JWXYS was the core CHM of cluster 1. JWXYS, DS, XF, and SZRT exhibited both of the reductions of H_2_O_2_-induced phosphorylation of p38 MAPK and p44/42 MAPK (Erk1/2) in human ARPE-19 retina cells. In cluster 2, SJHXT was the core CHM. SJHXT and NX showed both of the phosphorylation reductions. In cluster 3, GGT was the core CHM, and it reduced the phosphorylation of both MAPKs. In cluster 4, HQin was the core CHM, and it also reduced the phosphorylation of both MAPKs. Our study suggests that adjunctive CHM therapy may reduce diabetic retinopathy via antioxidant activity of the herbs and provides information on core CHM treatments for further scientific investigations or therapeutic interventions.

## INTRODUCTION

Type 2 diabetes (T2D) accounts for 90–95% of all cases of diabetes worldwide [1]. T2D patients have high levels of blood glucose and an impaired pancreatic β-cell function [2, 3]. Hyperglycemia damages several organs (e.g., blood vessels, heart, eyes, kidneys, and nerves) and causes cardiovascular and cerebrovascular diseases, retinopathy, nephropathy, neuropathy, and peripheral circulatory disorders. These complications are responsible for the morbidity and mortality of diabetic patients [4]. Diabetic retinopathy, the leading cause of acquired blindness [5], is associated with oxidative stress and inflammation [6, 7] and is one of the most common microvascular T2D complications.

The retina has high oxygen uptake and glucose oxidation rates and is susceptible to oxidative stress [8]. A higher level of mitochondrial superoxide has been observed when retina cells were incubated with high concentrations of glucose [9]. Superoxide is produced by glucose metabolism, and this reactive oxygen species (ROS) increases the oxidative stress; moreover, it is involved in the development of diabetic retinopathy [6, 7, 10]. T2D progression can be controlled by lifestyle changes [11] and pharmacological therapies, including hypoglycemic or antihyperglycemic, insulin-sensitizing, or insulin secretion-enhancing drugs [12–14]. However, these treatment regimens for the blood glucose control are frequently associated with side effects. Meta-analyses have shown that metformin-, sulfonylurea-, and thiazolidinedione-based therapies are associated with an increased risk of cardiovascular diseases and mortality [12–15]. Furthermore, long-term use of thiazolidinedione increases the risk of fractures, lower respiratory tract infections, and bladder cancer in T2D patients [14, 16, 17]. These findings have prompted to search for alternative and complementary therapies to improve the management of diabetes and its complications.

Chinese herbal medicine (CHM) is an important health care system in Taiwan [18, 19]. People in Taiwan can take regular antidiabetic drugs, CHMs, or both. CHM prescription patterns have been investigated for various diseases, such as childhood asthma [20], breast cancer [21], chronic kidney disease [22], diabetes [23], endometriosis [24], primary dysmenorrhea [25], schizophrenia [26], and Sjögren's syndrome [27]. Moreover, improvements in the survival rate, hyperglycemia, and/or inflammation, attributed to CHM treatments, have been reported in T2D patients [28–32]. There is, however, limited information about the effects of CHM on the occurrence of diabetic retinopathy in T2D patients. In this study, we used a population-based database to investigate the demographic characteristics, CHM prescription patterns, and CHM effects on the occurrence of diabetic retinopathy in T2D patients in Taiwan. In addition, we evaluated the protective effects of most commonly used CHM treatments on human retina cells in a hypoxic state in *in vitro* experiments.

## RESULTS

### Participants and their baseline characteristics

**Figure 1:**
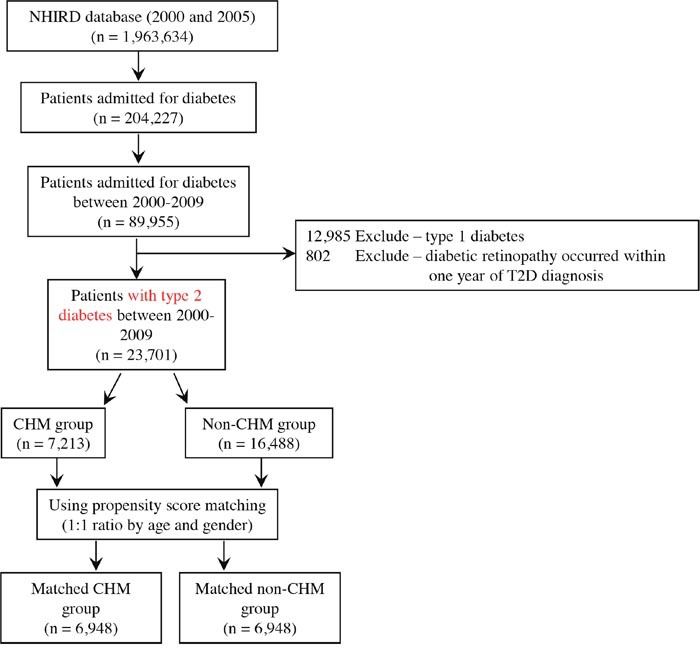
Enrollment of type 2 diabetes patients Patients with type 2 diabetes were identified for our study cohort after the exclusions listed above.

A total of 89,955 patients were identified in the Taiwan National Health Insurance (NHI) Research Database (NHIRD), who were admitted for diabetes treatment during the observation period (between 2000 and 2009) (Figure 1). Among those, 12,985 individuals with type 1 diabetes and 802 individuals with diabetic retinopathy that occurred within 1 year after diabetes had been diagnosed were excluded. In total, 23,701 patients diagnosed with T2D between 2000 and 2009 were included in our study cohort (Figure 1). Among these patients, 7,213 (30.4%) belonged to the CHM group, and 16,488 (69.6%) belonged to the non-CHM group (Figure 1 and Table 1).

**Table 1:** Baseline characteristics of total subjects and propensity score matched subjects with T2D according to CHM use

Characteristics	Total subjects		*p* value	Matched subjects		*p* value
	CHM group	Non-CHM group		CHM group	non-CHM group	
	N=7,213	N=16,488		N=6,948	N=6,948	
	N (%)	N (%)		N (%)	N (%)	
**Age**			***<0.001***			0.424
20-40 yrs	755 (10.47)	1,469 (8.91)		732 (10.54)	666 (9.59)	
40-50 yrs	1,589 (22.03)	3,044 (18.46)		1,490 (21.45)	1,460 (21.01)	
50-60 yrs	2,188 (30.33)	4,229 (25.65)		2,106 (30.31)	2,149 (30.93)	
60-70 yrs	1,574 (21.82)	3,221 (19.54)		1,513 (21.78)	1,520 (21.88)	
70-80 yrs	894 (12.39)	2,904 (17.61)		894 (12.87)	938 (13.50)	
≧80 yrs	213 (2.95)	1,621 (9.83)		213 (3.07)	215 (3.09)	
**Gender**			***<0.001***			0.973
Male	10,899 (66.10)	3,242 (44.95)		3,242 (46.66)	3,240 (46.63)	
Female	5,589 (33.90)	3,971 (55.05)		3,706 (53.34)	3,708 (53.37)	
**Chronic obstructive pulmonary disease**			***<0.001***			0.505
No	5,559 (77.07)	13,366 (81.07)		5,506 (79.25)	5,474 (78.79)	
Yes	1,654 (22.93)	3,122 (18.93)		1,442 (20.75)	1,474 (21.21)	
**Cerebrovascular disease**			***<0.001***			0.688
No	6,401 (88.74)	14,141 (85.77)		6,168 (88.77)	6,153 (88.56)	
Yes	812 (11.26)	2,347 (14.23)		780 (11.23)	795 (11.44)	
**Renal disease**			0.535			0.434
No	6,877 (95.34)	15,750 (95.52)		6,630 (95.42)	6,649 (95.70)	
Yes	336 (4.66)	738 (4.48)		318 (4.58)	299 (4.30)	
**Hyperlipidemia**			***<0.001***			***<0.001***
No	4,602 (63.80)	11,442 (69.40)		4,455 (64.12)	4,733 (68.12)	
Yes	2,611 (36.20)	5,046 (30.60)		2,493 (35.88)	2,215 (31.88)	
**Obesity**			0.249			0.845
No	7,156 (99.21)	16,380 (99.34)		6,894 (99.22)	6,896 (99.25)	
Yes	57 (0.79)	108 (0.66)		54 (0.78)	52 (0.75)	
**Alcohol-related illness**			0.018			0.911
No	7,173 (99.45)	16,349 (99.16)		6,908 (99.42)	6,907 (99.41)	
Yes	40 (0.55)	139 (0.84)		40 (0.58)	41 (0.59)	
**Hypertension**			***<0.001***			0.285
No	3,808 (52.79)	7,926 (48.07)		3,631 (52.26)	3,568 (51.35)	
Yes	3,405 (47.21)	8,562 (51.93)		3,317 (47.74)	3,380 (48.65)	
**Myocardial infarction**			***<0.001***			0.942
No	7,117 (98.67)	16,052 (97.36)		6,852 (98.62)	6,853 (98.63)	
Yes	96 (1.33)	436 (2.64)		96 (1.38)	95 (1.37)	
**Anti-hypertensives drug use**			<0.001			0.429
No	5265 (72.99)	12746 (77.30)		5146 (74.06)	5105 (73.47)	
Yes	1948 (27.01)	3742 (22.70)		1802 (25.94)	1843 (26.53)	
**Statin use**			<0.001			0.976
No	6571 (91.10)	15271 (92.62)		6353 (91.44)	6354 (91.45)	
Yes	642 (8.90)	1217 (7.38)		595 (8.56)	594 (8.55)	
**Insulin use**			0.085			0.178
No	7196 (99.76)	16426 (99.62)		6931 (99.76)	6938 (99.86)	
Yes	17 (0.24)	62 (0.38)		17 (0.24)	10 (0.14)	
**Income**			***<0.001***			***<0.001***
<NT20000	2,728 (37.82)	7,068 (42.87)		2,629 (37.84)	2,928 (42.14)	
NT20000∼NT30000	2,512 (34.83)	5,581 (33.85)		2,404 (34.60)	2,396 (34.48)	
NT30000∼NT40000	1,227 (17.01)	2,180 (13.22)		1,175 (16.91)	995 (14.32)	
>=NT40000	746 (10.34)	1,659 (10.06)		740 (10.65)	629 (9.05)	
**Urbanization level**			***<0.001***			***0.004***
1	3,112 (43.14)	6,830 (41.42)		3,003 (43.22)	2,975 (42.82)	
2	1,797 (24.91)	3,950 (23.96)		1,727 (24.86)	1,674 (24.09)	
3	663 (9.19)	1,411 (8.56)		641 (9.23)	592 (8.52)	
4	623 (8.64)	1,558 (9.45)		600 (8.64)	573 (8.25)	
5	1,018 (14.11)	2,739 (16.61)		977 (14.06)	1,134 (16.32)	

*p* values were obtained by chi-square test. *p* value (*p* < 0.05) was highlighted in bold italic.

T2D, type 2 diabetes; CHM, Chinese herbal medicine; N, number; NT, new Taiwan dollars.

Urbanization level: 1 indicates the hightest level of urbanization and 5 is the lowest level.

The comorbidities include chronic obstructive pulmonary disease (ICD-9-CM: 490–496), cerebrovascular disease (ICD-9-CM: 430–438), renal disease (ICD-9-CM: 582, 583–583.7, 585, 586, and 588), hyperlipidemia (ICD-9-CM: 272), obesity (ICD-9-CM: 278 and 278.01), alcohol-related illness (ICD-9-CM: 303, 305, 305.01, 305.02, 305.03, and V11.3), hypertension (ICD-9-CM: 401-405), and myocardial infarction (ICD-9-CM: 410 and 412). These comorbidities are identified before the T2D diagnosis.

As shown in Table 1, there were significantly different frequency distributions in age, gender, comorbidities (chronic obstructive pulmonary disease, cerebrovascular disease, hyperlipidemia, hypertension, and myocardial infarction), medications (antihypertensive drug and statin use), income, and urbanization level between the CHM and non-CHM groups (*P* < 0.05). To minimize the bias in the estimated effects (i.e., group difference), these two groups were age- and gender-matched at a 1:1 ratio (Table 1), and we found significantly different frequency distributions for hyperlipidemia, income, and urbanization level (*P* < 0.05). The CHM group was characterized by more cases of hyperlipidemia, a higher income, and a higher level of urbanization.

### Effect of CHM on the occurrence of diabetic retinopathy among type 2 diabetes patients

With regard to the effect of CHM on the occurrence of diabetic retinopathy among the T2D patients, the CHM use tended to be associated with a reduced hazard ratio compared with that among the CHM non-users (Table 2; overall hazard ratio: 0.88; 95% confidence interval: 0.70–1.10; *P* = 0.244). The same trend was also found when the subjects were stratified into male and female subgroups (Table 2). In particular, this subgroup analysis showed that the use of CHM was associated with a protective effect in the female subgroup (hazard ratio: 0.56; 95% confidence interval: 0.36–0.86; *P* = 0.008).

**Table 2:** Hazard ratios (95% CI) for diabetic retinopathy when T2D patients were stratified by gender

CHM user (Ref: non-CHM user)	Hazard ratio (95% CI)	*p* value
**Overall**	0.88 (0.70-1.10)	0.244
**Gender**		
Male	0.85 (0.52-1.83)	0.511
Female	0.56 (0.36-0.86)	***0.008***

CHM, Chinese herbal medicine; Ref: Reference; T2D, type 2 diabetes; CI, confidence interval.

*Models adjusted for age, comorbidities, anti-hypertensives drug before T2D, and statin before T2D.

Cox's proportional hazards model and Fine & Grays’ model were applied in this analysis.

*p* value (*p* < 0.05) was highlighted in bold italic.

**Figure 2:**
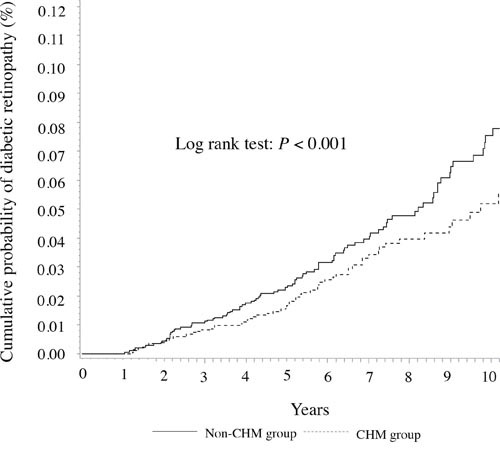
Cumulative probability of diabetic retinopathy in female patients with T2D according to the CHM use

The cumulative probability of diabetic retinopathy in the female subgroups with T2D (according to the CHM use) is shown in Figure 2. In the female subgroups, the cumulative probability of diabetic retinopathy was lower among the CHM users than that among the CHM non-users (*P* < 0.001, log-rank test).

### Most commonly used CHM products and their effects on H_2_O_2_-induced oxidative stress in human ARPE-19 retina cells

The top 10 Chinese herbal formulations and single herbs used by the female subgroup are listed in Table 3, along with their compositions. The follow-up person-years were calculated as the interval between the T2D diagnosis and the end of the study. Based on the percentage of users, Jia-Wei-Xiao-Yao-San (JWXYS; 52.9%) was the most commonly prescribed herbal formula, followed by Shu-Jing-Huo-Xue-Tang (SJHXT; 45.1%), Ge-Gen-Tang (GGT; 43.7%), Shao-Yao-Gan-Cao-Tang (SYGCT; 38.7%), Chuan-Xiong-Cha-Tiao-San (CXCTS; 38.0%), Yin-Qiao-San (YQS; 35.4%), Xue-Fu-Zhu-Yu-Tang (XFZYT; 34.6%), Ma-Xing-Shi-Gan-Tang (MXSGT; 33.8%), Liu-Wei-Di-Huang-Wan (LWDHW; 33.3%), and Suan-Zao-Ren-Tang (SZRT; 30.9%).

**Table 3:** Ten most commonly used herbal formulas and single herbs for female patients with T2D

Formulas	Pin-yin name (shorten name)	Chinese name	Number of herbs	Composition (Pin-yin name (latin name; botanical plant name))	User number	Percentage of user number	Frequency of prescription	Person-year	Average drug dose per day (g)	Average duration for prescription (days)
**Total**					2068	100	96735	12847.3	12.2	7.2
**Herbal formula^a^**					2064	99.8	93106	12831.2	9.4	7.2
	**Jia-Wei-Xiao-Yao-San (JWXYS)**	加味逍遙散	10	**Dang-Gui** (*Radix Angelicae Sinensi; Angelica sinensis (Oliv.) Diels*), **Bai-Shao** (*Radix Paeoniae Alba*; *Paeonia lactiflora Pall*.), **Fu-Ling** (*Poria*; *Wolfiporia extensa (Peck) Ginns*), **Bai-Zhu** (*Rhizoma Atractylodis Macrocephalae*; *Atractylodes macrocephala Koidz*.), **Chai-Hu** (*Radix Bupleuri*; *Bupleurum falcatum L*.), **Mu-Dan-Pi** (*Cortex Moutan*; *Moutan officinalis (L.) Lindl. & Paxton*), **Zhi-Zi** (*Fructus Gardeniae*; *Gardenia jasminoides J.Ellis*), **Gan-Cao** (*Radix Glycyrrhizae Preparata*; *Glycyrrhiza uralensis Fisch*.), **Bo-He** (*Herba Menthae Haplocalycis*; *Mentha arvensis L*.), **Sheng-Jiang** (*Rhizoma Zingiberis Recens*; *Zingiber officinale Roscoe*)	1095	52.9	8669	7016.2	4.4	8.7
	**Shu-Jing-Huo-Xue-Tang (SJHXT)**	舒經活血湯	17	**Dang-Gui** (*Radix Angelicae Sinensi*; *Angelica sinensis (Oliv.) Diels*)**, Bai-Shao** (*Radix Paeoniae Alba*; *Paeonia lactiflora Pall*.), **Chuan-Xiong** (*Rhizoma Chuanxiong*; *Ligusticum sinense Oliv*.), **Di-Huang** (*Radix Rehmanniae*; *Rehmannia glutinosa (Gaertn.) DC*.), **Tao-Ren** (*Semen Persicae*; *Prunus persica (L.) Batsch*), **Bai-Zhu** (*Rhizoma Atractylodis*; *Atractylodes macrocephala Koidz*.), **Fu-Ling** (*Poria*; *Wolfiporia extensa (Peck) Ginns*), **Niu-Xi** (*Radix Achyranthis Bidentatae*; *Achyranthes bidentata Blume*), **Wei-Ling-Xian** (*Radix Clematidis*; *Clematis chinensis Osbeck*), **Han-Fang-Ji** (*Radix Stephaniae Tetrandrae*; *Stephania tetrandra S.Moore*), **Qiang-Huo** (*Rhizoma seu Radix Notopterygii*; *Notopterygium forbesii var. oviforme (Shan) H.T. Chang*), **Fang-Feng** (*Radix Saposhnikoviae*; *Saposhnikovia divaricata (Turcz.) Schischk*.), **Long-Dan-Cao** (*Radix Gentianae*; *Gentiana lutea L*.), **Bai-Zhi** (*Radix Angelicae Dahuricae*; *Angelica dahurica (Hoffm.) Benth. & Hook.f. ex Franch. & Sav*.), **Chen-Pi** (*Pericarpium Citri Reticulatae*; *Citrus reticulata Blanco*), **Gan-Cao** (*Radix Glycyrrhizae Preparata*; *Glycyrrhiza uralensis Fisch*.), **Sheng-Jiang** (*Rhizoma Zingiberis Recens*; *Zingiber officinale Roscoe*)	932	45.1	5137	6279.1	4	7.4
	**Ge-Gen-Tang (GGT)**	葛根湯	7	**Ge-Gen** (*Radix Puerariae*; *Pueraria lobata (Willd.) Ohwi*), **Ma-Huang** (*Herba Ephedrae*; *Ephedra vulgaris Rich*.), **Gui-Zhi** (*Cinnamomi ramulus*; *Cinnamomum cassia (L.) J.Presl*), **Bai-Shao** (*Radix Paeoniae Alba*; *Paeonia lactiflora Pall*.), **Sheng-Jiang** (Rhizoma Zingiberis Recens; Zingiber officinale Roscoe), **Da-Zao** (*Fructus Jujube*; *Ziziphus jujuba Mill*.), **Gan-Cao** (*Radix Glycyrrhizae Preparata*; *Glycyrrhiza uralensis Fisch*.)	904	43.7	4658	5994.8	4.2	6.5
	**Shao-Yao-Gan-Cao-Tang (SYGCT)**	芍藥甘草湯	2	**Bai-Shao** (Radix Paeoniae Alba; Paeonia lactiflora Pall.), **Gan-Cao** (Radix Glycyrrhizae Preparata; Glycyrrhiza uralensis Fisch.)	801	38.7	3738	5373.8	3	6.4
	**Chuan-Xiong-Cha-Tiao-San (CXCTS)**	川芎茶調散	10	**Bo-He** (*Herba Menthae Haplocalycis*; *Mentha arvensis L*.), **Chuan-Xiong** (*Rhizoma Chuanxiong*; *Ligusticum sinense Oliv*.), **Bai-Zhi** (*Radix Angelicae Dahuricae*; *Angelica dahurica (Hoffm.) Benth. & Hook.f. ex Franch. & Sav*.), **Qiang-Huo** (*Rhizoma seu Radix Notopterygii*; *Notopterygium forbesii var. oviforme (Shan) H.T. Chang*), **Xi-Xin** (*Herba cum Radix Asari*; *Asarum sieboldii Miq*.), **Xiang-Fu** (*Rhizoma Cyperi*; *Cyperus rotundus L*.), **Jing-Jie** (*Herba Schizonepetae*; *Schizonepeta tenuifolia (Benth.) Briq*.), **Fang-Feng** (*Radix Saposhnikoviae*; *Saposhnikovia divaricata (Turcz.) Schischk*.), **Gan-Cao** (*Radix Glycyrrhizae Preparata*; *Glycyrrhiza uralensis Fisch*.), **Lu-Cha** (*Folium Camelliae Sinensis*; *Camellia sinensis (L.) Kuntze*)	786	38	4687	5323.6	4.3	6.2
	**Yin-Qiao-San (YQS)**	銀翹散	10	**Jin-Yin-Hua** (*Flos Lonicerae*; *Lonicera japonica Thunb*.), **Lian-Qiao** (*Fructus Forsythiae*; *Forsythia suspensa (Thunb.) Vahl*), **Jie-Geng** (*Radix Platycodi*; *Platycodon grandiflorus (Jacq.) A.DC*.), **Niu Bang Zi** (*Fructus Arctii*; *Arctium lappa L*.), **Bo-He** (*Herba Menthae Haplocalycis*; *Mentha arvensis L*.), **Dan-Dou-Chi** (*Semen Sojae Praeparatum*; *Glycine max (L.) Merr*.), **Dan-Zhu-Ye** (*Herba Lophatheri*; *Lophatherum gracile Brongn*.), **Jing-Jie** (*Herba Schizonepetae*; *Schizonepeta tenuifolia (Benth.) Briq*.), **Lu-Gen** (*Rhizoma Phragmitis*; *Phragmites communis Trin*.), **Gan-Cao** (*Radix Glycyrrhizae Preparata*; *Glycyrrhiza uralensis Fisch*.)	733	35.4	3490	4969.8	3.8	5.9
	**Xue-Fu-Zhu-Yu-Tang (XFZYT)**	血府逐瘀湯	11	**Tao-Ren** (*Semen Persicae*; *Prunus persica (L.) Batsch*), **Hong-Hua** (*Flos Carthami*; *Carthamus tinctorius L*.), **Dang-Gui** (*Radix Angelicae Sinensi*; *Angelica sinensis (Oliv.) Diels*), **Chuan-Xiong** (*Rhizoma Chuanxiong*; *Ligusticum sinense Oliv*.), **Chi-Shao** (Radix Paeoniae Rubra; *Paeonia lactiflora Pall*.), **Chuan-Niu-Xi** (*Radix Cyathulae*; *Achyranthes bidentata Blume*), **Chai-Hu** (*Radix Bupleuri*; *Bupleurum falcatum L*.), **Jie-Geng** (*Radix Platycodi*; *Platycodon grandiflorus (Jacq.) A.DC*.), **Zhi-Shi** (*Fructus Aurantii Immaturus*; *Citrus aurantium L*.), **Sheng-Di-Huang** (*Radix Rehmanniae*; *Rehmannia glutinosa (Gaertn.) DC*.), **Gan-Cao** (Radix Glycyrrhizae Preparata; Glycyrrhiza uralensis Fisch.)	716	34.6	3563	4771.1	3.6	8.2
	**Ma-Xing-Shi-Gan-Tang (MXSGT)**	麻杏石甘湯	4	**Ma-Huang** (*Herba Ephedrae*; *Ephedra sinica Stapf*), **Xing-Ren** (*Semen Armeniacae*; *Prunus armeniaca L*.), **Shi-Gao** (*Gypsum Fibrosum*), **Gan-Cao** (*Radix Glycyrrhizae Preparata*; *Glycyrrhiza uralensis Fisch*.)	699	33.8	2819	4755.3	3.7	6
	**Liu-Wei-Di-Huang-Wan (LWDHW)**	六味地黄丸	6	**Shu-Di-Huang** (*Radix Rehmanniae Preparata*; *Rehmannia glutinosa (Gaertn.) DC*.), **Shan-Zhu-Yu** (*Fructus Corni*; *Cornus officinalis Siebold & Zucc*.), **Shan-Yao** (*Rhizoma Dioscoreae*; *Dioscorea opposita Thunb*.), **Fu-Ling** (*Poria*; *Wolfiporia extensa (Peck) Ginns*), **Mu-Dan-Pi** (*Cortex Moutan*; *Moutan officinalis (L.) Lindl. & Paxton*), **Ze-Xie** (*Rhizoma Alismatis*; *Alisma plantago-aquatica L*.)	688	33.3	3475	4662	4.4	8.1
	**Suan-Zao-Ren-Tang (SZRT)**	酸棗仁湯	5	**Suan-Zao-Ren** (*Semen Zizyphi Spinosae*; *Ziziphus jujuba Mill*.), **Fu-Ling** (*Poria*; *Wolfiporia extensa (Peck) Ginns*), **Zhi-Mu** (*Rhizoma Anemarrhenae*; *Anemarrhena asphodeloides Bunge*.), **Gao-Ben** (*Rhizoma Ligustici*; *Ligusticum sinense Oliv*.), **Gan-Cao** (*Radix Glycyrrhizae Preparata*; *Glycyrrhiza uralensis Fisch*.)	638	30.9	3486	4204.3	3.9	7.7
**Single herbs^a^**					2031	98.2	77320	12617	4	7.3
	**Yan-Hu-Suo (YHS)**	延胡索	1	**Yan-Hu-Suo** (*Rhizoma Corydalis*; *Corydalis yanhusuo (Y.H.Chou & Chun C.Hsu) W.T.Wang ex Z.Y.Su & C.Y.Wu*)	994	48.1	5999	6572.8	1.1	6.9
	**Ge-Gen (GG)**	葛根	1	**Ge-Gen** (*Radix Puerariae*; *Pueraria lobata (Willd.) Ohwi*)	871	42.1	5016	5737.6	1.3	7.3
	**Huang-Qin (HQin)**	黄芩	1	**Huang-Qin** (*Radix Scutellariae*; *Scutellaria baicalensis Georgi*)	830	40.1	4604	5475.6	1.1	7
	**Dan-Shen (DS)**	丹參	1	**Dan-Shen** (*Radix Salviae Miltiorrhizae*; *Salvia miltiorrhiza Bunge*)	814	39.4	5297	5270.5	1.2	8.9
	**Jie-Geng (JG)**	桔梗	1	**Jie-Geng** (*Radix Platycodi*; *Platycodon grandiflorus (Jacq.) A.DC*.)	792	38.3	4359	5135.2	1	6.3
	**Bei-Mu (BM)**	貝母	1	**Bei-Mu** (*Bulbus Fritillariae Cirrhosae*; *Fritillaria cirrhosa D.Don*)	765	37	4900	5038	1.1	6.7
	**Tian-Hua-Fen (THF)**	天花粉	1	**Tian-Hua-Fen** (*Radix Trichosanthis*; *Trichosanthes kirilowii Maxim*.)	746	36.1	4173	4928.7	1.2	7.9
	**Niu-Xi (NX)**	牛膝	1	**Niu-Xi** (*Radix Achyranthis Bidentatae*; *Achyranthes bidentata Blume*)	659	31.9	3182	4482.9	1.1	7.7
	**Xiang-Fu (XF)**	香附	1	**Xiang-Fu** (*Rhizoma Cyperi*; *Cyperus rotundus L*.)	657	31.8	3327	4347.2	1	7.2
	**Huang-Qi (HQ)**	黃耆	1	**Huang-Qi** (*Radix Astragali*; *Astragalus membranaceus (Fisch.) Bunge*)	653	31.6	3219	4273.4	1.3	8.1

*Sorted by user number. T2D, type 2 diabetes.

^a^Information are obtained from the websites (http://www.americandragon.com/index.htm; http://old.tcmwiki.com/; http://www.shen-nong.com/eng/front/index.html; http://www.ipni.org/; http://www.theplantlist.org/).

Of the 10 most common single herbs, Yan-Hu-Suo (YHS; 48.1%) was most frequently prescribed, followed by Ge-Gen (GG; 42.1%), Huang-Qin (HQin; 40.1%), Dan-Shen (DS; 39.4%), Jie-Geng (JG; 38.3%), Bei-Mu (BM; 37.0%), Tian-Hua-Fen (THF; 36.1%), Niu-Xi (NX; 31.9%), Xiang-Fu (XF; 31.8%), and Huang-Qi (HQ; 31.6%).

It is possible that the loss of vision caused by diabetic retinopathy is associated with oxidative damage inflicted on retinal pigment epithelial cells [8, 33]. Studies have shown that H_2_O_2_-induced oxidative stress increased p38 mitogen-activated protein kinase (MAPK) and extracellular signal-regulated kinase (ERK) phosphorylation in human retina cells [34, 35]. Therefore, to explore the potential signaling pathways involved in the protective CHM effects against H_2_O_2_-induced damage, we examined the phosphorylation status of p38 MAPK and p44/42 MAPKs (Erk1/2) in retina cells.

**Figure 3:**
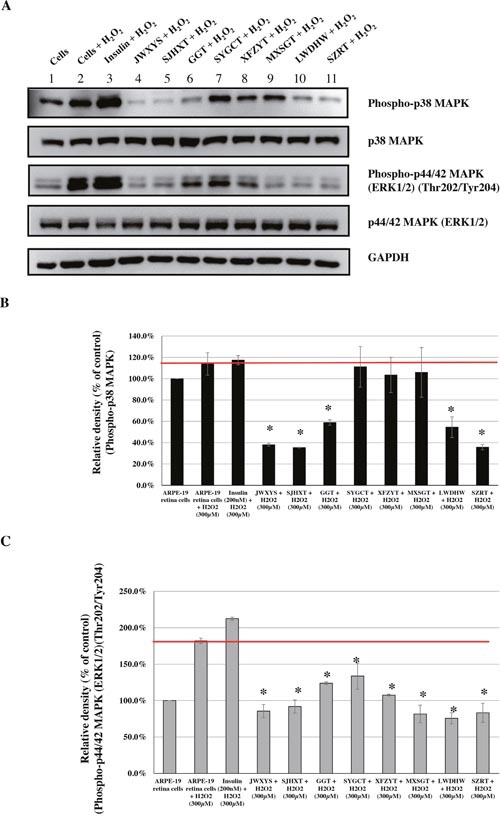
Effects of CHMs (JWXYS, SJHXT, GGT, SYGCT, XFZYT, MXSGT, LWDHW, and SZRT) on H_2_O_2_-treated ARPE-19 cells Cells treated with insulin (200 nM) were used as controls. The insulin- and CHM-treated cells were then incubated with an H_2_O_2_ (300 μM) solution. Western blot analysis was performed by staining membranes with anti-phospho-p38 MAPK, anti-p38 MAPK, anti-phospho-p44/42 MAPK, anti-p44/42 MAPK, and anti-GAPDH antibodies. **(A)** Western blot analysis of phospho-p38 MAPK, p38 MAPK, phospho-p44/42 MAPK, p44/42 MAPK, and GAPDH protein expression. **(B)** The ratio of phospho-p38 MAPK to p38 MAPK in various treatment groups versus that in untreated cells. *P < 0.05. **(C)** The ratio of phospho-p44/42 MAPKs to p44/42 MAPKs in various treatment groups versus that in untreated cells. *P < 0.05.

**Figure 4:**
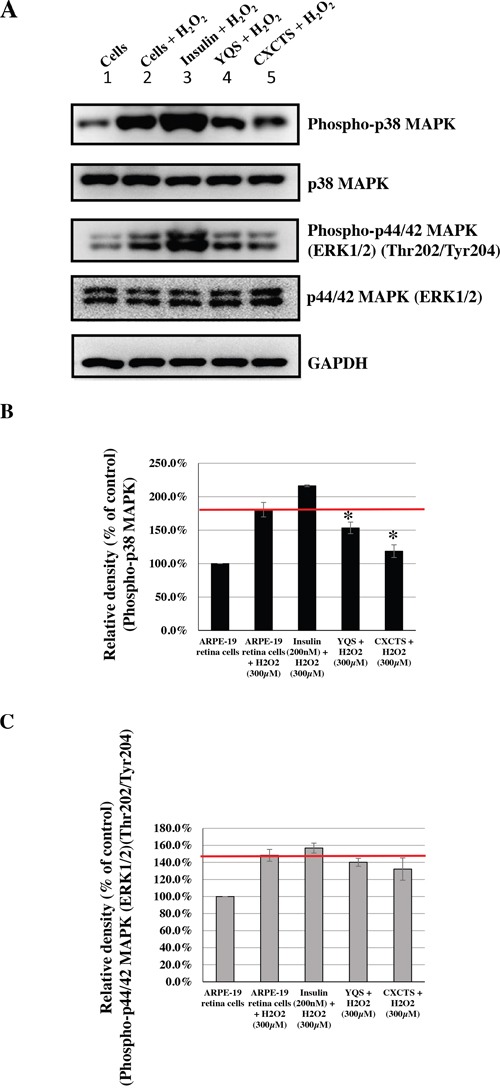
Effects of CHMs (YQS and CXCTS) on H_2_O_2_-treated ARPE-19 cells Cells treated with insulin (200 nM) were used as controls. The insulin- and CHM-treated cells were then incubated with an H_2_O_2_ (300 μM) solution. Western blot analysis was performed by staining membranes with anti-phospho-p38 MAPK, anti-p38 MAPK, anti-phospho-p44/42 MAPK, anti-p44/42 MAPK, and anti-GAPDH antibodies. **(A)** Western blot analysis of phospho-p38 MAPK, p38 MAPK, phospho-p44/42 MAPK, p44/42 MAPK, and GAPDH protein expression. **(B)** The ratio of phospho-p38 MAPK to p38 MAPK in various treatment groups versus that in untreated cells. *P < 0.05. **(C)** The ratio of phospho-p44/42 MAPKs to p44/42 MAPKs in various treatment groups versus that in untreated cells.

**Figure 5:**
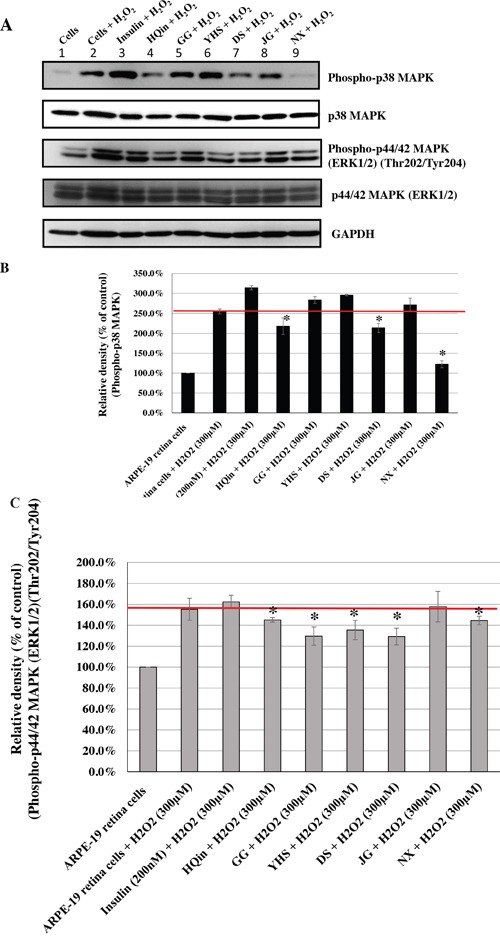
Effects of CHMs (HQin, GG, YHS, DS, JG, and NX) on H_2_O_2_-treated ARPE-19 cells Cells treated with insulin (200 nM) were used as controls. The insulin- and CHM-treated cells were then incubated with an H_2_O_2_ (300 μM) solution. Western blot analysis was performed by staining membranes with anti-phospho-p38 MAPK, anti-p38 MAPK, anti-phospho-p44/42 MAPK, anti-p44/42 MAPK, and anti-GAPDH antibodies. **(A)** Western blot analysis of phospho-p38 MAPK, p38 MAPK, phospho-p44/42 MAPK, p44/42 MAPK, and GAPDH protein expression. **(B)** The ratio of phospho-p38 MAPK to p38 MAPK in various treatment groups versus that in untreated cells. *P < 0.05. **(C)** The ratio of phospho-p44/42 MAPKs to p44/42 MAPKs in various treatment groups versus that in untreated cells. *P < 0.05.

**Figure 6:**
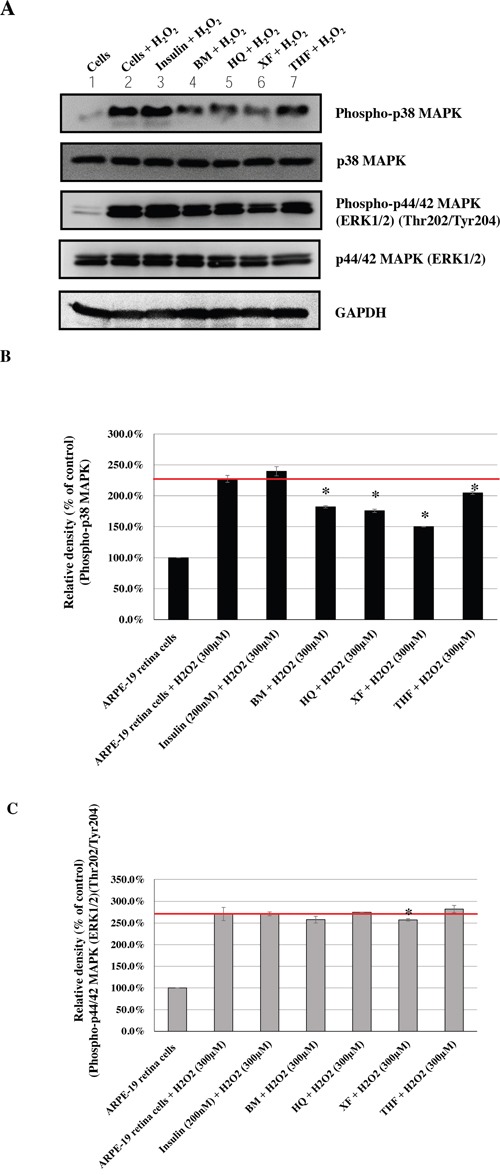
Effects of CHMs (BM, THF, HQ, and XF) on H_2_O_2_-treated ARPE-19 cells Cells treated with insulin (200 nM) were used as controls. The insulin- and CHM-treated cells were then incubated with an H_2_O_2_ (300 μM) solution. Western blot analysis was performed by staining membranes with anti-phospho-p38 MAPK, anti-p38 MAPK, anti-phospho-p44/42 MAPK, anti-p44/42 MAPK, and anti-GAPDH antibodies. **(A)** Western blot analysis of phospho-p38 MAPK, p38 MAPK, phospho-p44/42 MAPK, p44/42 MAPK, and GAPDH protein expression. **(B)** The ratio of phospho-p38 MAPK to p38 MAPK in various treatment groups versus that in untreated cells. *P < 0.05. **(C)** The ratio of phospho-p44/42 MAPKs to p44/42 MAPKs in various treatment groups versus that in untreated cells. *P < 0.05.

Based on the information about the most commonly prescribed CHM products, we evaluated their effects on the phosphorylation of p38 MAPK and p44/42 MAPKs (Erk1/2), caused by H_2_O_2_-induced oxidative stress. Human ARPE-19 retina cells were treated with these most commonly used CHM products at the indicated concentrations (Figures 3, 4, 5, 6). Insulin was used as the control. The CHM- and insulin-treated cells were then incubated with an H_2_O_2_ solution. As shown in Figures 3A, 4A, 5A, and 6A, the human retina cells treated with H_2_O_2_ showed increased phosphorylation of p38 MAPK and p44/42 MAPKs (Erk1/2) when compared with the untreated cells (Lanes 1 and 2). In addition, there were slight increases in the phosphorylation of p38 MAPK and p44/42 MAPKs (Erk1/2) in the insulin + H_2_O_2_-treated cells compared with that in the H_2_O_2_-treated cells (Figures 3A, 4A, 5A, and 6A, Lanes 2 and 3).

Interestingly, among the top 10 herbal formulations, the H_2_O_2_-induced phosphorylation of p38 MAPK was reduced by JWXYS, SJHXT, GGT, LWDHW, SZRT, YQS, and CXCTS compared with that in the H_2_O_2_-treated cells (*P* < 0.05; Figures 3B and 4B). The H_2_O_2_-induced phosphorylation of p44/42 MAPKs (Erk1/2) was reduced by JWXYS, SJHXT, GGT, SYGCT, XFZYT, MXSGT, LWDHW, and SZRT compared with that in the H_2_O_2_-treated cells (*P* < 0.05; Figure 3C).

Among the top 10 single herbs, the H_2_O_2_-induced phosphorylation of p38 MAPK was reduced by HQin, DS, NX, BM, HQ, XF, and THF when compared with that in the H_2_O_2_-treated cells (*P* < 0.05; Figures 5B and 6B). The H_2_O_2_-induced phosphorylation of p44/42 MAPKs (Erk1/2) was reduced by HQin, GG, YHS, DS, NX, and XF when compared with that in the H_2_O_2_-treated cells (*P* < 0.05; Figures 5C and 6C).

### CHM network for female patients with T2D

**Figure 7:**
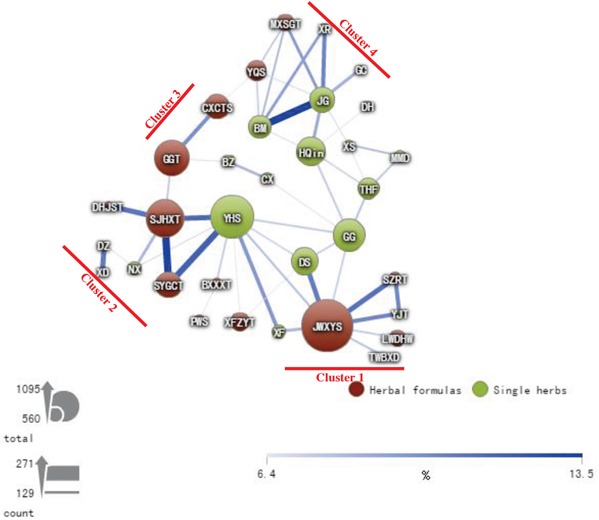
CHM network for female patients with T2D The connection lines between CHMs represent user numbers for CHM combinations. The connection between CHMs is more important when the connection line is thicker and darker. The size of the circle represents the frequency of prescriptions for each CHM.

Our results suggested that the cumulative probability of diabetic retinopathy was lower for the CHM users than that for the CHM non-users in the female subgroups (Table 2 and Figure 2). To explore the CHM network and core treatments prescribed for these female patients with T2D, CHM combinations and their constituted networks were identified from the Taiwan NHIRD (Table 4 and Figure 7). The CHM network was analyzed for these patients and found to present complicated relationships among CHM products. During the study period, 1,828 female patients with T2D used CHMs, and 26,977 prescriptions were made by traditional Chinese medicine (TCM) doctors. Among the prescriptions, the top six CHM combinations are shown in Table 4. In addition, CHM network analysis was performed for these patients (Figure 7). Four clusters were found among the top CHM combinations, and each cluster was constituted by a core CHM and its important combinations. JWXYS was the core CHM of cluster 1, which was the largest CHM cluster. In this cluster, JWXYS, DS, XF, and SZRT reduced the H_2_O_2_-induced phosphorylation of both p38 MAPK and p44/42 MAPKs (Erk1/2). In cluster 2, SJHXT was the core CHM, and SJHXT and NX were important CHMs, which both reduced the H_2_O_2_-induced phosphorylation of p38 MAPK and p44/42 MAPKs (Erk1/2). In cluster 3, GGT was the core CHM and the only CHM that reduced the H_2_O_2_-induced phosphorylation of both p38 MAPK and p44/42 MAPKs (Erk1/2). In cluster 4, HQin was the core CHM and the only CHM that reduced the H_2_O_2_-induced phosphorylation of p38 MAPK and p44/42 MAPKs (Erk1/2). The following interconnections were also observed among these clusters: cluster 4 was connected to cluster 1, cluster 1 was connected to cluster 2, and cluster 2 was connected to cluster 3 (Figure 7).

**Table 4:** Six most commonly used combinations for female patients with T2D

CHM combinations	User number	Percentage of user number	Frequency of prescription	Person-year	Average drug dose per day (g)	Average duration for prescription (days)
**Total**	1828	100	26977	11449	5.7	7.4
**Shu-Jing-Huo-Xue-Tang (SJHXT) and Shao-Yao-Gan-Cao-Tang (SYGCT)**	271	14.8	774	1873	6.5	6.8
**Jie-Geng (JG) and Bei-Mu (BM)**	270	14.8	821	1761	2.1	6.3
**Shao-Yao-Gan-Cao-Tang (SYGCT) and Yan-Hu-Suo (YHS)**	237	13	640	1606	4.2	6.4
**Shu-Jing-Huo-Xue-Tang (SJHXT) and Yan-Hu-Suo (YHS)**	227	12.4	648	1489	5.2	7
**Jia-Wei-Xiao-Yao-San (JWXYS) and Suan-Zao-Ren-Tang (SZRT)**	221	12.1	702	1490	7.5	8.1
**Jia-Wei-Xiao-Yao-San (JWXYS) and Dan-Shen (DS)**	212	11.6	827	1478	5.7	10.4

*Sorted by user number.

^a^Information are obtained from the websites (http://www.americandragon.com/index.htm; http://old.tcmwiki.com/; http://www.shen-nong.com/eng/front/index.html; http://www.ipni.org/; http://www.theplantlist.org/).

CHM, Chinese herbal medicine; T2D, type 2 diabetes.

## DISCUSSION

This study showed that adjunctive CHM treatment might reduce the occurrence of diabetic retinopathy among female patients with T2D. The herbal formulas, single herbs, and their combinations, most commonly used by these patients, were identified and evaluated in terms of their protective effects against oxidative stress and inflammation in ARPE-19 retina cells. In addition, the core CHMs, CHM clusters, and CHM cluster interconnections were identified and presented as a consensus CHM prescription pattern for the target disease. This is valuable information for TCM doctors and researchers that can be used in the clinic and in further investigations. To our knowledge, this is the first population-based pharmacoepidemiological study that focused on the CHM effects on diabetic retinopathy in T2D patients. Our results suggested that adjunctive CHM treatment may reduce the occurrence of diabetic retinopathy by reducing oxidative stress in human retina cells.

Our results showed that among the female patients, the cumulative probability of diabetic retinopathy was lower in CHM users than in CHM non-users. We also found that there were significantly different CHM prescription patterns and networks between female and male patients (Supplementary Figure 1). For the female patients, JWXYS was the core CHM in cluster 1. Among CHMs from this cluster, JWXYS, DS, XF, and SZRT reduced the H_2_O_2_-induced phosphorylation of both p38 MAPK and p44/42 MAPKs (Erk1/2) in retina cells *in vitro*. Furthermore, cells treated with JWXYS, DS, and XF also showed reduced ROS levels by flow cytometry, compared with those in H_2_O_2_-treated cells. JWXYS is composed of 10 single herbs and has been prescribed for many different conditions, including anorexia, dry eyes, headache, hot flashes, irregular menstruation, nervousness, night sweating, and palpitations, as well as for hepatoprotection [36–40]. Furthermore, JWXYS has been used for many different diseases, including hypertension with T2D, systemic lupus erythematosus, urolithiasis, chronic hepatitis B infection, liver cancer, colon cancer, breast cancer, sleep disorders, major depressive disorders, and dementia [28, 41–48]. Among the 10 single herbs from this formulation, Dang-Gui (*Angelica sinensis* (Oliv.) Diels) and Bai-Shao (*Paeonia lactiflora* Pall.) are the two major herbs. Dang-Gui (*A. sinensis* (Oliv.) Diels) contains two major natural compounds, ferulic acid and coniferyl ferulate [49], exhibiting antioxidant and anti-inflammatory activities [50, 51]. Bai-Shao (*P. lactiflora* Pall.) contains paeoniflorin and albiflorin [52, 53], both showing antioxidant and anti-inflammatory activities [52, 54]. Therefore, there are at least four natural compounds with antioxidant and anti-inflammatory activities, ferulic acid, coniferyl ferulate, paeoniflorin, and albiflorin, in JWXYS (cluster 1). We examined their effects on ROS levels in retina cells by flow cytometry and found that cells treated with these natural compounds demonstrated reduced ROS levels compared with those in H_2_O_2_-treated cells. DS (*Salvia miltiorrhiza* Bunge) contains diterpenoid quinones, hydrophilic phenolic acids, and essential oils and shows antioxidant, neuroprotective, antifibrotic, anti-inflammatory, and antineoplastic activities [55, 56]. XF (*Cyperus rotundus* L.) is a medicinal herb traditionally used to treat various clinical conditions, including diarrhea, diabetes, pyresis, inflammation, malaria, as well as stomach and bowel disorders [57]. XF (*C. rotundus* L.) contains essential oils, phenolic acids, ascorbic acid, and flavonoids in tubers and rhizomes and exhibits anti-inflammatory activities [58].

In cluster 2, SJHXT was the core CHM. SJHXT and NX were found to be important CHMs that reduced the H_2_O_2_-induced phosphorylation of both p38 MAPK and p44/42 MAPKs (Erk1/2). SJHXT is composed of 17 single herbs and has been prescribed for hundreds years for the treatment of chronic pain syndromes, including fractures [59], breast cancer [47], and prostate cancer [60]. Its pharmacological activities include anti-inflammatory and analgesic effects [61], antihypersensitivity activity [62], and the ability to increase blood circulation [63]. Among the 17 single herbs in this formulation, Dang-Gui (*A. sinensis* (Oliv.) Diels) and Bai-Shao (*P. lactiflora* Pall.) are the two major single herbs. Therefore, similar to JWXYS, ferulic acid, coniferyl ferulate, paeoniflorin, and albiflorin are the major components of SJHXT (cluster 2), which demonstrated ROS-reducing effects in retina cells treated with H_2_O_2_. NX (*Achyranthes bidentata* Blume) is often used in TCM for the treatment of arthritis since it possesses anti-inflammatory properties. The main active component is an oleanane-type saponin, which was shown to inhibit interleukin-1β-induced nuclear factor-κB activation in rat chondrocytes [64].

In cluster 3, GGT was the core CHM and the only CHM in this cluster that reduced the H_2_O_2_-induced phosphorylation of both p38 MAPK and p44/42 MAPKs (Erk1/2) in retina cells. GGT is a traditional Chinese medicinal formula composed of seven single herbs. There are seven pharmacologically active natural compounds, puerarin, daidzin, daidein, paeoniflorin, albiflorin, liquiritin, and liquiritigenin, detected in GGT [65]. This formula is widely used as a treatment for a common cold and migraine, as well as to improve the symptoms of gastrointestinal and respiratory disorders [66–68]. Among the seven single herbs, GG (*Pueraria lobata* (Willd.) Ohwi), Bai-Shao (*P. lactiflora* Pall.), and Gan-Cao (*Glycyrrhiza uralensis* Fisch.) are the three major herbs. GG (*P. lobata* (Willd.) Ohwi) contains an isoflavonoid glycoside with hypotensive activity, which has shown excellent clinical results for the treatment of hypertension [69], as well as puerarin, daidzin, and daidein [70]. Puerarin, the major active ingredient of GG, has been shown to exert significant protective effects against diabetic retinopathy in rats by regulating the expression of factors involved in angiogenesis [70]. As previously described, Bai-Shao (*P. lactiflora* Pall.) contains paeoniflorin and albiflorin [52, 53]. Gan-Cao (*G. uralensis* Fisch.) contains glycyrrhizin, glycyrrhizic acid, liquiritin, and liquiritigenin [71] and exhibits anti-inflammatory activities [72, 73]. Therefore, there are at least nine known pharmacologically active natural compounds, puerarin, daidzin, daidein, paeoniflorin, albiflorin, glycyrrhizin, glycyrrhizic acid, liquiritin, and liquiritigenin, in the herbal formula GGT (cluster 3). Among these nine natural compounds, treatment with glycyrrhizin and liquiritigenin (without H_2_O_2_) resulted in detectable fluorescence, suggesting that the color of glycyrrhizin and liquiritigenin may interfere with the detection of intracellular ROS production by flow cytometry. Thus, these two natural compounds are not suitable for the detection of the protective effect against H_2_O_2_-induced ROS using the flow cytometry method. We, therefore, investigated the effects of the rest of the natural compounds. The results suggested that these natural compounds from GGT reduced the cellular ROS levels compared with those in H_2_O_2_-treated cells.

In cluster 4, HQin was the core CHM and the only CHM that reduced the H_2_O_2_-induced phosphorylation of both p38 MAPK and p44/42 MAPKs (Erk1/2). Since treatment with HQin alone (without H_2_O_2_) resulted in detectable fluorescence, this single herb and its relevant natural compounds are not suitable for the detection of intracellular ROS by flow cytometry. HQin is composed of *Scutellaria baicalensis* Georgi and affects glucose-stimulated insulin secretion and β-cell proliferation. It also induces anti-apoptotic effects on vascular endothelial cells, resulting in the prevention of diabetes-associated microvascular complications [74, 75]. In addition, HQin attenuates oxidative stress and inflammation [76, 77]. Our *in vitro* experiments showed that HQin reduced the H_2_O_2_-induced phosphorylation of both p38 MAPK and p44/42 MAPKs (Erk1/2) in ARPE-19 retina cells.

To our knowledge, this is the first population-based pharmacoepidemiological study focused on the effect of CHM use on the occurrence of diabetic retinopathy. By using the Taiwan NHIRD, we were able to investigate the demographic characteristics, cumulative probability of retinopathy, CHM prescription patterns, and the networks of T2D patients. We also evaluated the CHM effects on human retina cells *in vitro*. This investigation may provide the scientific basis or therapeutic directions for the prevention of diabetic retinopathy.

In conclusion, diabetic retinopathy is one of the microvascular complications of T2D and the leading cause of acquired blindness. Our results suggest that adjunctive CHM treatment may reduce the occurrence of diabetic retinopathy owing to antioxidant activities of the herbs.

## MATERIALS AND METHODS

### Study population

This study was a population-based, case-control study. The study subjects were selected from the Longitudinal Health Insurance Database (LHID2000 and LHID2005). Individuals with diabetes (ICD-9-CM: 250) were identified between the years 2000 and 2009. Patients with type 1 diabetes or diabetic retinopathy that occurred within 1 year of T2D diagnosis were excluded (Figure 1). After applying these criteria, 23,701 study subjects were included in the study cohort. Subjects who took CHM products for more than 28 days within the first year of T2D were defined as CHM users (*n* = 7,213; Figure 1). These CHM users continued to use CHM products during this study period (between the index date and study endpoint). The index date was defined as the date by which 28 days of CHM treatment had been achieved. Subjects with no record of CHM treatment were defined as CHM non-users during this study period (*n* = 16,488; Figure 1). The date of death, withdrawal from the NHI program, or follow-up termination (December 31, 2012) was considered the study endpoint. The propensity score matching method was used to match the CHM users and non-users at a 1:1 ratio. After matching these two groups for age and gender, CHM users and non-users were selected (Figure 1 and Table 1). All data for each individual were coded, and therefore we could not obtain their informed consent. This study was evaluated and approved by the Institutional Review Board of the China Medical University Hospital.

### Chinese herbal medicines

Single-herb preparations are obtained from plants, animals, or mineral materials. These are mixed to create a formulation. Herbal formulations contain a combination of 2–17 herbs (Table 3), which are prepared by experienced TCM doctors. These formulations have been used since ancient China. The single herbs and herbal formulations from this study were produced by good manufacturing practice-certified TCM manufacturers based in Taiwan. These manufacturers included Sun Ten Pharmaceutical Co., Ltd., Shang Chang Pharmaceutical Co., Ltd., Chuang Song Zong Pharmaceutical Co., Ltd., KO DA Pharmaceutical Co., Ltd., and Kaiser Pharmaceutical Co., Ltd. For each herbal product, the number of users, percentage of users, frequency of prescription, person-years, average drug dose per day, and average duration of prescription were collected and calculated from the day of T2D diagnosis through the study endpoint (Table 3).

### Cell culture and CHM treatment

Human retinal pigmented epithelium cells (ARPE-19 cell line; American Type Culture Collection CRL-2302) were maintained in Dulbecco's modified Eagle's medium supplemented with 10% fetal bovine serum, 100 U/mL penicillin, 100 μg/mL streptomycin, and 2 mM L-glutamine (Gibco, Thermo Fisher Scientific, Waltham, MA, USA). A human insulin solution (catalog number I9278) and hydrogen peroxide solution (catalog number 18304) were purchased from Sigma–Aldrich (St. Louis, MO, USA). ARPE-19 cells were treated with insulin (200 nM; Supplementary Figures 2 and 3), herbal formulas (1 mg/mL), and single herbs (1 mg/mL) for 4 h (Table 3 and Figures 3, 4, 5, 6). The insulin- and CHM-treated ARPE-19 cells were then incubated with the H_2_O_2_ solution (300 μM; Supplementary Figures 2 and 3) for 30 min. The treated cells were lysed in RIPA buffer (catalog number 89900, Thermo Fisher Scientific, Rockford, IL, USA) with protease inhibitors (complete EDTA-free protease inhibitors, catalog number 11873580001, Sigma–Aldrich) and a phosphatase inhibitor (catalog number 88667, Thermo Fisher Scientific), then subjected to 12% sodium dodecyl sulfate polyacrylamide gel electrophoresis, and transferred to polyvinylidene difluoride membranes (Millipore, Billerica, MA, USA). To perform Western blot analysis, the membranes were incubated with primary antibodies overnight at 4°C. The primary antibodies included anti-phospho-p38 MAPK (Thr180/Tyr182; D3F9, **catalog number 4511)**, anti-p38 MAPK **(catalog number 9212)**, anti-phospho-p44/42 MAPK (Erk1/2) (Thr202/Tyr204)(**catalog number 4370)**, and anti-p44/42 MAPK (Erk1/2) (**catalog number 4695)** antibodies **from Cell Signaling Technology, Inc. (Beverly, MA, USA)** and an anti-glyceraldehyde 3-phosphate dehydrogenase (GAPDH) antibody (catalog number 10494-1-AP) from Proteintech Group, Inc. (Rosemont, IL, USA). The membranes were then incubated with alkaline phosphatase-conjugated secondary antibodies (Sigma–Aldrich). Signals were visualized using a chemiluminescence kit (Chemicon), following the manufacturer's protocol.

In addition, detection of intracellular ROS production was performed using flow cytometry (Supplementary Figures 4–7). ARPE-19 retina cells were pretreated with insulin, CHMs, or natural compounds for 18 h. The treated cells were then stained with 5 μM 2′,7′-dichlorofluorescin diacetate (DCFH-DA) for 30 min, followed by incubation with H_2_O_2_ (300 μM) for 5 min to induce the intracellular ROS generation. DCFH-DA fluorescence intensities were then measured by flow cytometry at an excitation wavelength of 488 nm and an emission wavelength of 535 nm (FACSCanto™ flow cytometry system; BD Biosciences, San Jose, CA, USA). The relative fluorescence intensity of ROS is expressed as fluorescence intensity of treated cells versus that of the untreated control.

### Statistical analysis

Demographic data, including age, gender, comorbidities, medications, income, and urbanization levels were analyzed for both CHM users and non-users. Each categorical variable is presented as the number or percentage of patients, and chi-squared tests were performed (Table 1). Comorbidity was also considered as a study covariate based on the disease history prior to T2D diagnosis (Table 1). We identified the following comorbidities: chronic obstructive pulmonary disease (ICD-9-CM: 490–496), cerebrovascular disease (ICD-9-CM: 430–438), renal disease (ICD-9-CM: 582, 583–583.7, 585, 586, and 588), hyperlipidemia (ICD-9-CM: 272), obesity (ICD-9-CM: 278 and 278.01), alcohol-related illness (ICD-9-CM: 303, 305, 305.01, 305.02, 305.03, and V11.3), hypertension (ICD-9-CM: 401–405), and myocardial infarction (ICD-9-CM: 410 and 412) (Table 1). Urbanization levels in Taiwan are divided into five strata, according to Taiwan National Health Research Institutes publications, with level 1 referring to the most urbanized communities and level 5 referring to the least urbanized communities. The Cox's proportional hazards model was applied to evaluate the effect of CHM on the occurrence of diabetic retinopathy among T2D patients (Table 2). The Kaplan–Meier method and log-rank test were employed to estimate the cumulative probability of diabetic retinopathy between CHM users and non-users (Figure 2). The top 10 most commonly used herbal formulations and single herbs are shown according to the number of users for each CHM (Table 3). The top six most commonly used CHM combinations are shown according to the number of users for each combination (Table 4). The social network analysis of SAS Visual Analytics was applied to explore the CHM network and core treatments for female patients with T2D from the Taiwan NHIRD (http://blogs.sas.com/content/sascom/2014/02/19/exploring-social-networks-with-sas-visual-analytics/; Table 4 and Figure 7). All *P*-values of less than 0.05 were considered significant. Data manipulation and statistical analyses were performed using the Statistical Analysis System software (version 9.3; SAS Institute, Cary, NC, USA).

## SUPPLEMENTARY MATERIALS FIGURES



## Abbreviations

ARPE-19 cells: Adult Retinal Pigment Epithelial cell line-19 cells
T2D: type 2 diabetes
CHM: Chinese herbal medicine
CI: confidence interval.
