# Supplementary material for: Effects of Chinese herbal medicines on the occurrence of diabetic retinopathy in type 2 diabetes patients and protection of ARPE-19 retina cells by inhibiting oxidative stress
Source: Oncotarget. 2017 Jun 29;8(38):63528–50. doi: 10.18632/oncotarget.18846 (PMC5609941; doi:10.18632/oncotarget.18846)
Supplement: Supplementary file 1 [file oncotarget-08-63528-s001.pdf]

Effects of Chinese herbal medicines on the occurrence of diabetic retinopathy in type 2 diabetes patients and protection of ARPE-19 retina cells by inhibiting oxidative stress

SUPPLEMENTARY MATERIALS

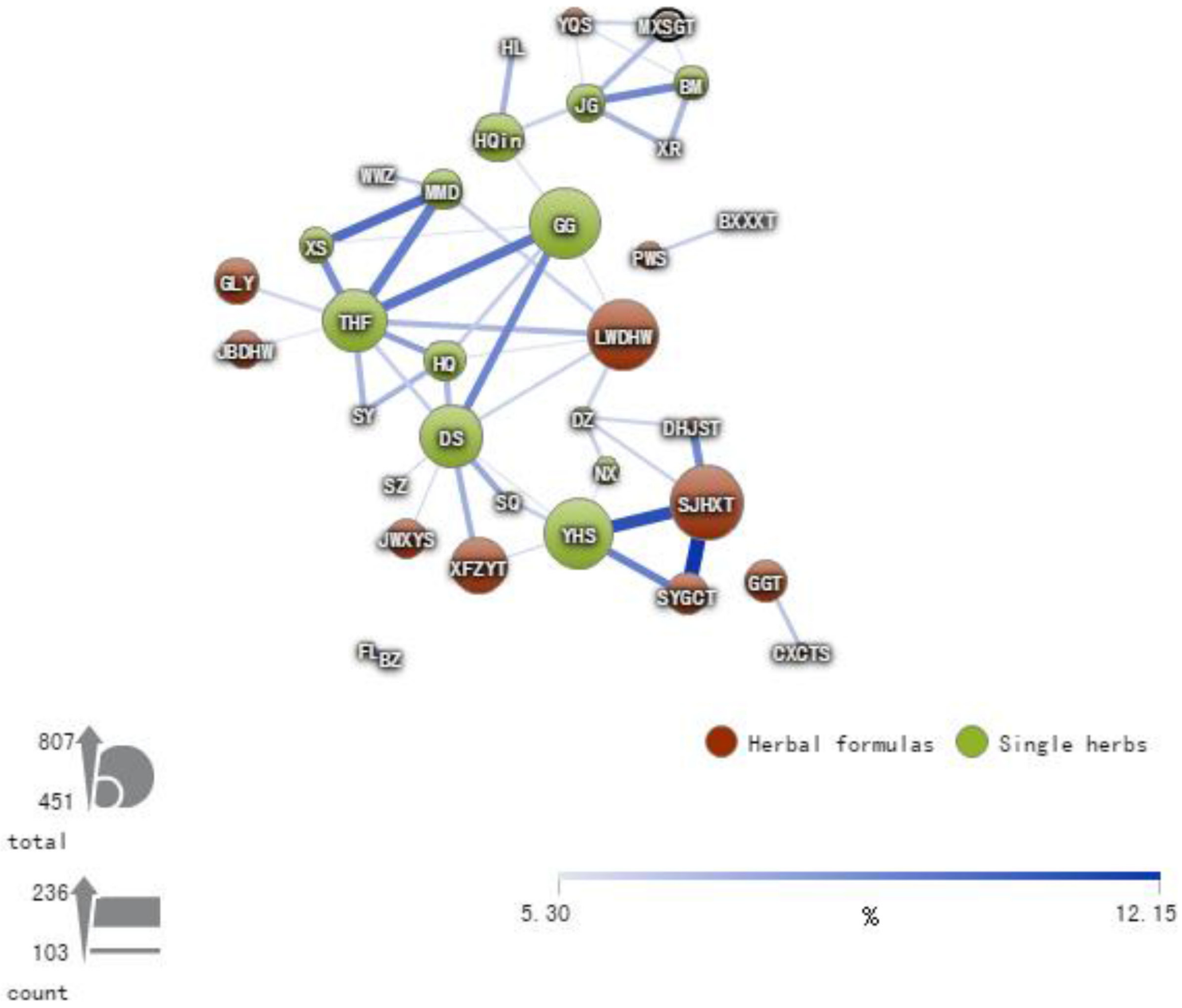

Supplementary Figure 1: CHM network for male patients with T2D. The connection line between CHMs represents user numbers for the CHM combinations. The connection between CHMs is more important when the connection line is thicker and darker. The size of circle represents frequency of prescription of each CHM. The larger circle means higher frequencies of prescription. The red color is for herbal formula; the green color is for single herb.

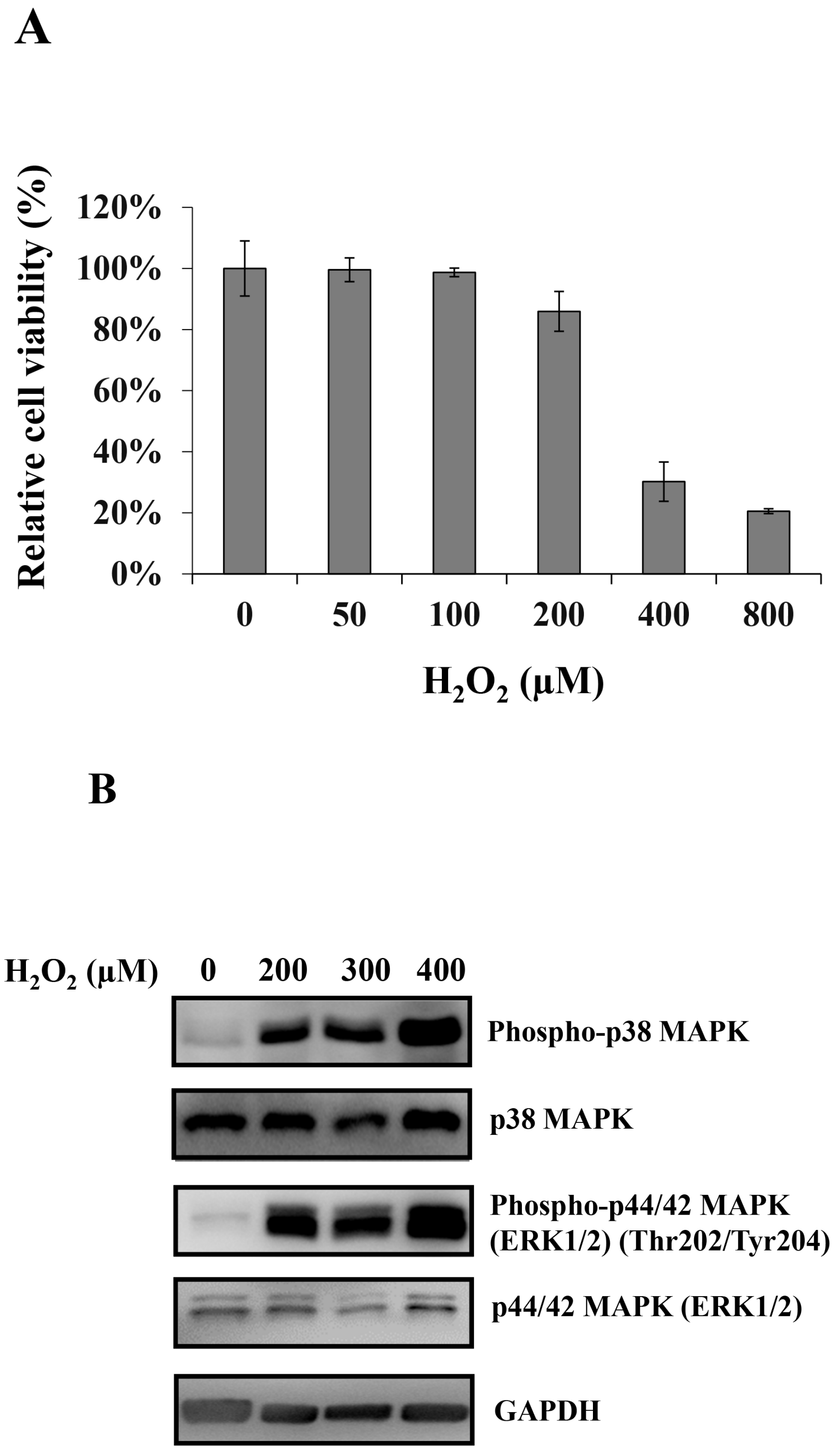

Supplementary Figure 2: Hydrogen peroxide (H_2O_2) induced ARPE-19 cell death and activated mitogen-activated protein kinase (MAPK). (A) Cell viability of ARPE-19 cells following H_2O_2 exposure were detected by WST-1 assay. ARPE-19 cells were treated with or without various H_2O_2 concentrations (50-800 µM) for 24h. Cell viability was measured by the WST-1 assay. Relative cell viability is expressed as percentage of the un-treated control, and each value represents the mean±SEM of three independent experiments. **(B)** ARPE-19 cells were treated with or without various H_2O_2 concentrations (200-400 µm) for 30 min and cell lysates were prepared. Western blot analysis and staining with anti-phosphor-p38 MAPK, anti-p38 MAPK, anti-phospho-p44/42 MAPK, anti- p44/42 MAPK, and anti-GAPDH antibodies was then performed.

(*Continued*)

C

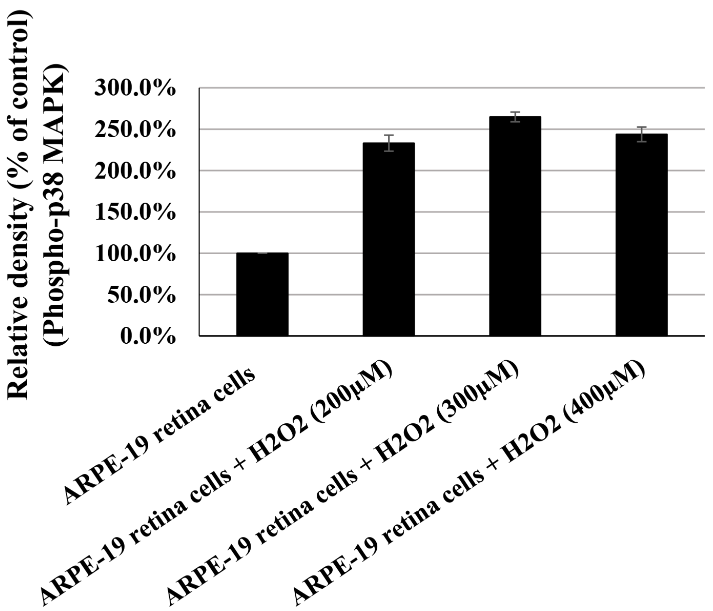

D

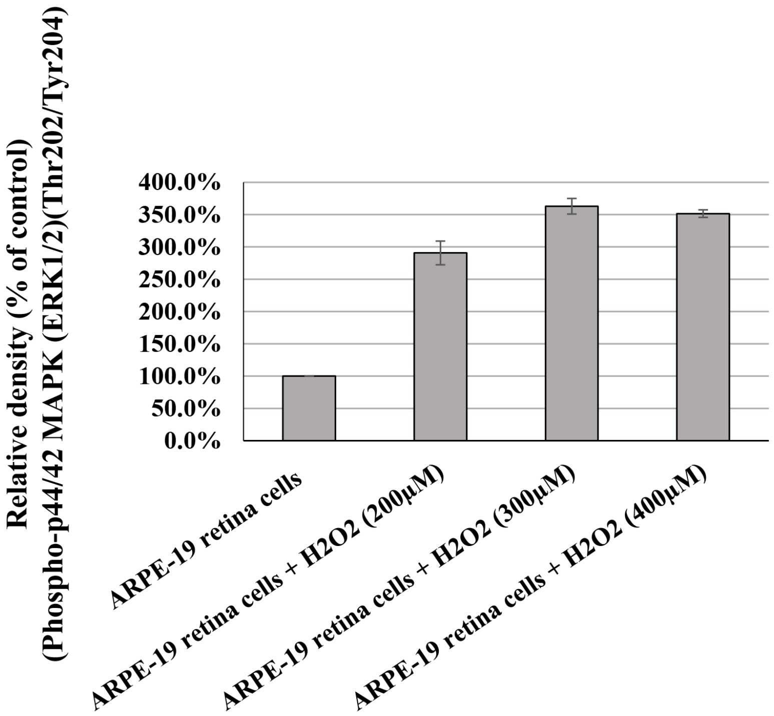

Supplementary Figure 2 (*Continued*): (C) The ratio of phospho-p38 MAPK to p38 MAPK in various groups [(phospho-p38 MAPK / p38 MAPK)group/(phospho-p38 MAPK / p38 MAPK)cells only × 100%]. **(D)** The ratio of phospho-p44/42 MAPK to p44/42 MAPK in various groups [(phospho-p44/42 MAPK / p44/42 MAPK)group/(phospho-p44/42 MAPK / p44/42 MAPK)cells only × 100%].

A

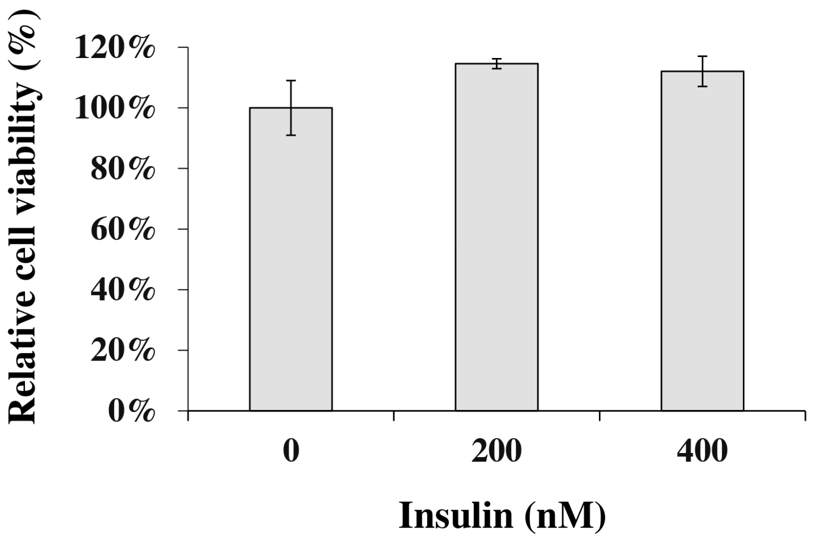

B

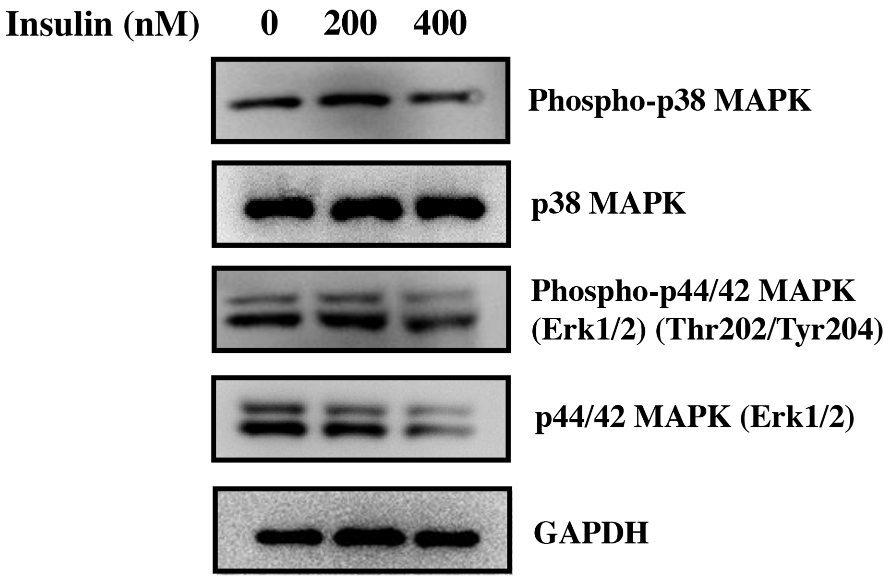

Supplementary Figure 3: Effects of insulin on ARPE-19 cell viability and mitogen-activated protein kinase (MAPK) phosphorylations of p38 MAPK and p44/42 MAPK proteins. **(A)** Cell viability of ARPE-19 cells following insulin exposure were detected by WST-1 assay. ARPE-19 cells were treated with or without various insulin concentrations (200-400 nM) for 24h. Cell viability was measured by the WST-1 assay. Relative cell viability is expressed as percentage of the un-treated control, and each value represents the mean±SEM of three independent experiments. **(B)** ARPE-19 cells were treated with or without various insulin concentrations (200 and 400 nM, respectively) for 4 h and cell lysates were prepared. Western blot analysis and staining with anti-phospho-p38 MAPK, anti-p38 MAPK, anti-phospho-p44/42 MAPK, anti-p44/42 MAPK, and anti-GAPDH antibodies was then performed.

(*Continued*)

C

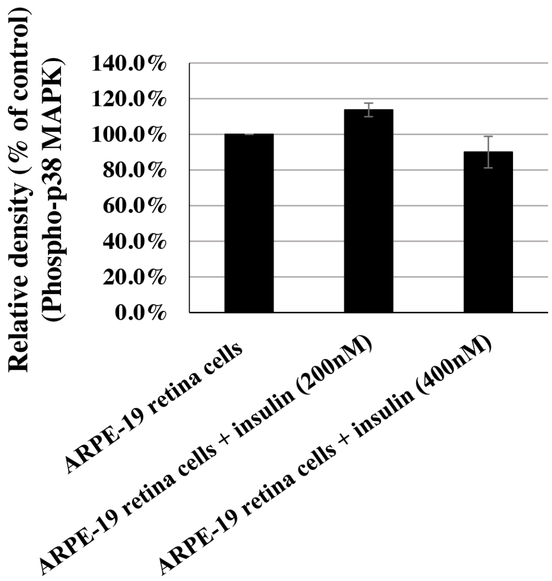

D

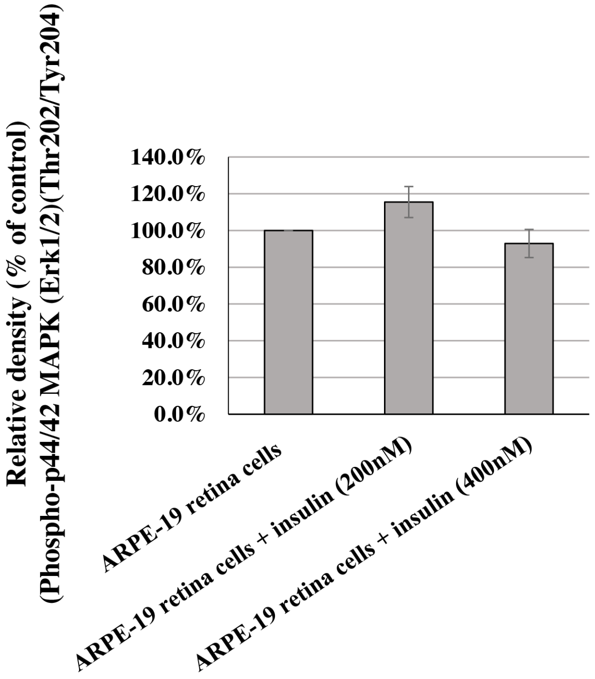

Supplementary Figure 3 (*Continued*): (C) The ratio of phospho-p38 MAPK to p38 MAPK in various groups [(phospho-p38 MAPK / p38 MAPK)group/(phospho-p38 MAPK / p38 MAPK)cells only × 100%]. **(D)** The ratio of phospho-p44/42 MAPK to p44/42 MAPK in various groups [(phospho-p44/42 MAPK / p44/42 MAPK)group/(phospho-p44/42 MAPK / p44/42 MAPK)cells only × 100%].

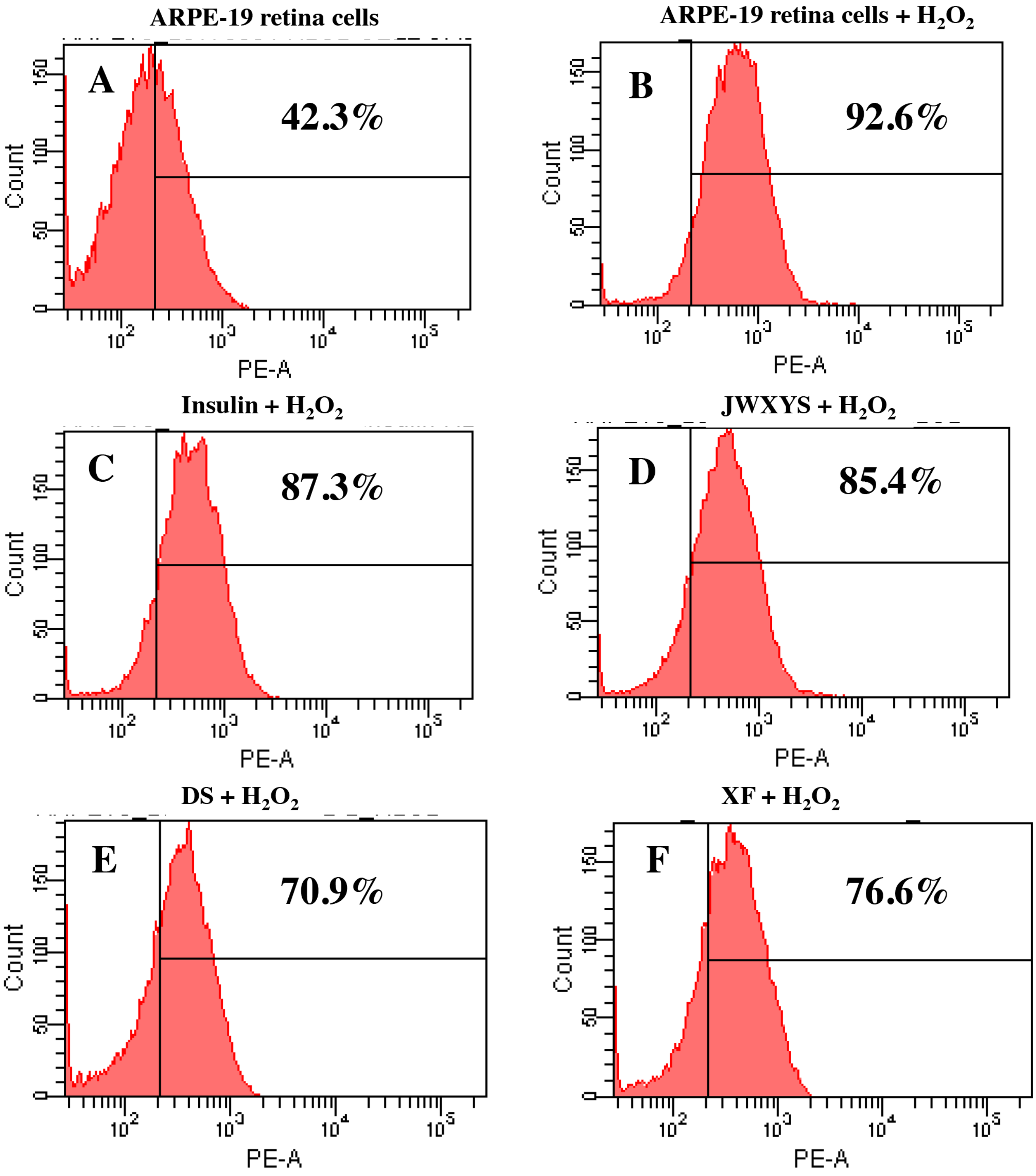
ARPE-19 retina cells
A
42.3%
ARPE-19 retina cells + H2O2
B
92.6%
Insulin + H2O2
C
87.3%
JWXYS + H2O2
D
85.4%
DS + H2O2
E
70.9%
XF + H2O2
F
76.6%
Count
PE-A

(Continued)

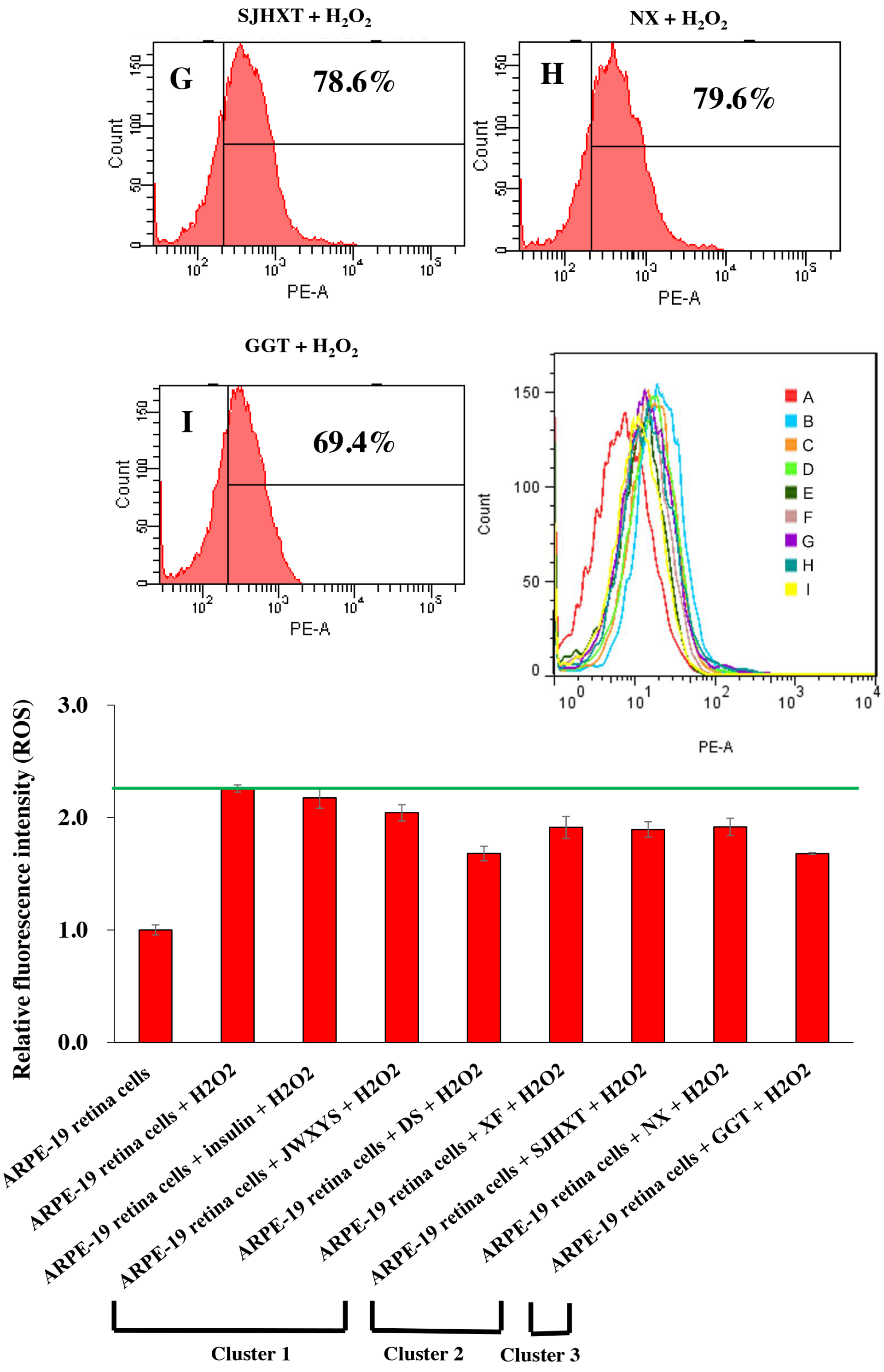

Supplementary Figure 4: Effects of CHM on hydrogen peroxide (H_2O_2)- induced ROS production in ARPE-19 retina cells. ARPE-19 retina cells were pretreated with or without insulin and CHM, respectively for 18 h. The treated cells were then stained with 5 μM DCFH-DA for 30 min and subsequently incubated with H_2O_2 (300 μM) for 5 min to induce the intracellular ROS generation. DCF fluorescence intensities were then measured by flow cytometry with an excitation wavelength of 488 nm and an emission wavelength of 535 nm, respectively (BD FACSCanto™ flow cytometry system, BD Biosciences, San Jose, CA). Relative fluorescence intensity (ROS) is expressed as relative intensity of the treated cells when compared with the un-treated control. Each value represents the mean±SEM of three independent experiments.

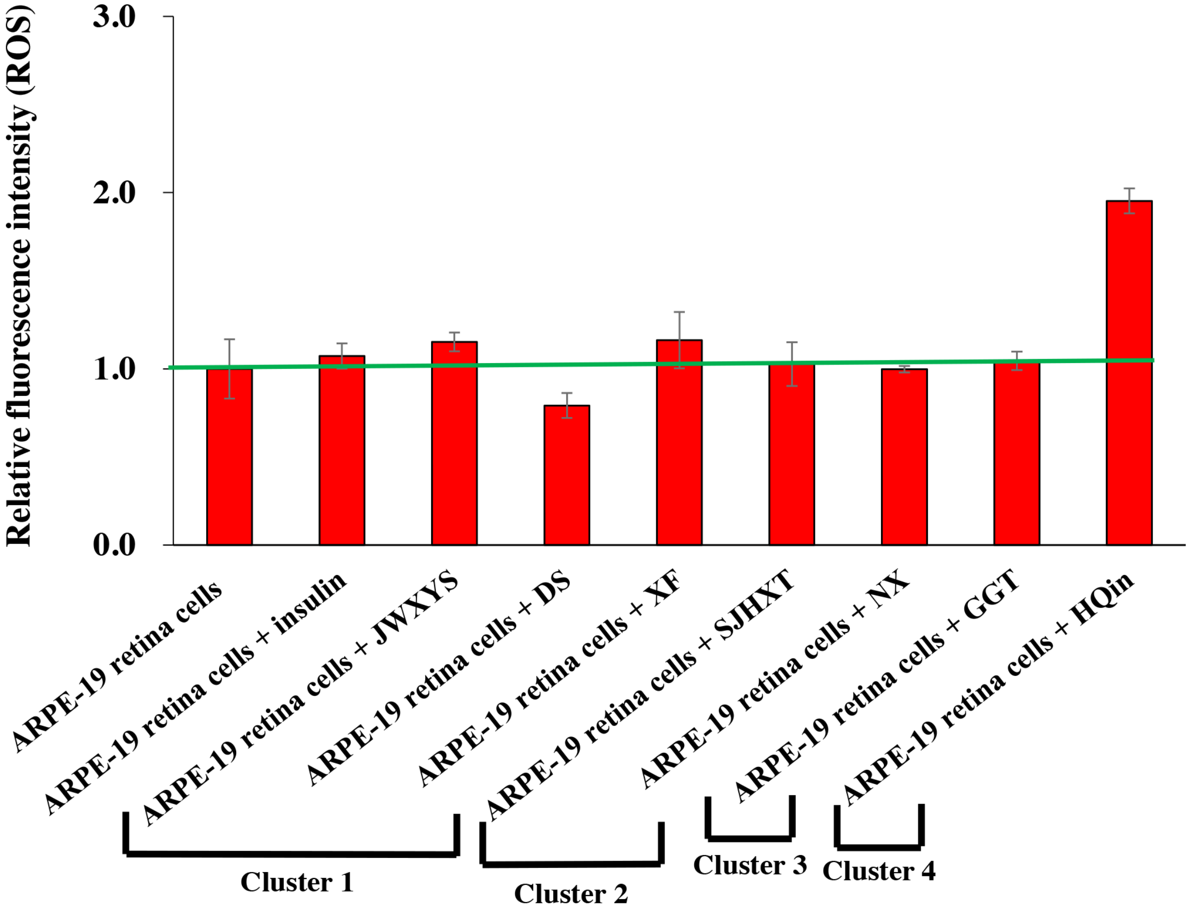

Supplementary Figure 5: Effects of CHM on intracellular ROS level in ARPE-19 retina cells. ARPE-19 retina cells were pretreated with or without insulin and CHM, respectively for 18 h. The treated cells were then stained with 5 μM DCFH-DA for 30 min. DCF fluorescence intensities were then measured by flow cytometry with an excitation wavelength of 488 nm and an emission wavelength of 535 nm, respectively (BD FACSCanto™ flow cytometry system, BD Biosciences, San Jose, CA). Relative fluorescence intensity (ROS) is expressed as relative intensity of the treated cells when compared with the un-treated control. Each value represents the mean±SEM of three independent experiments.

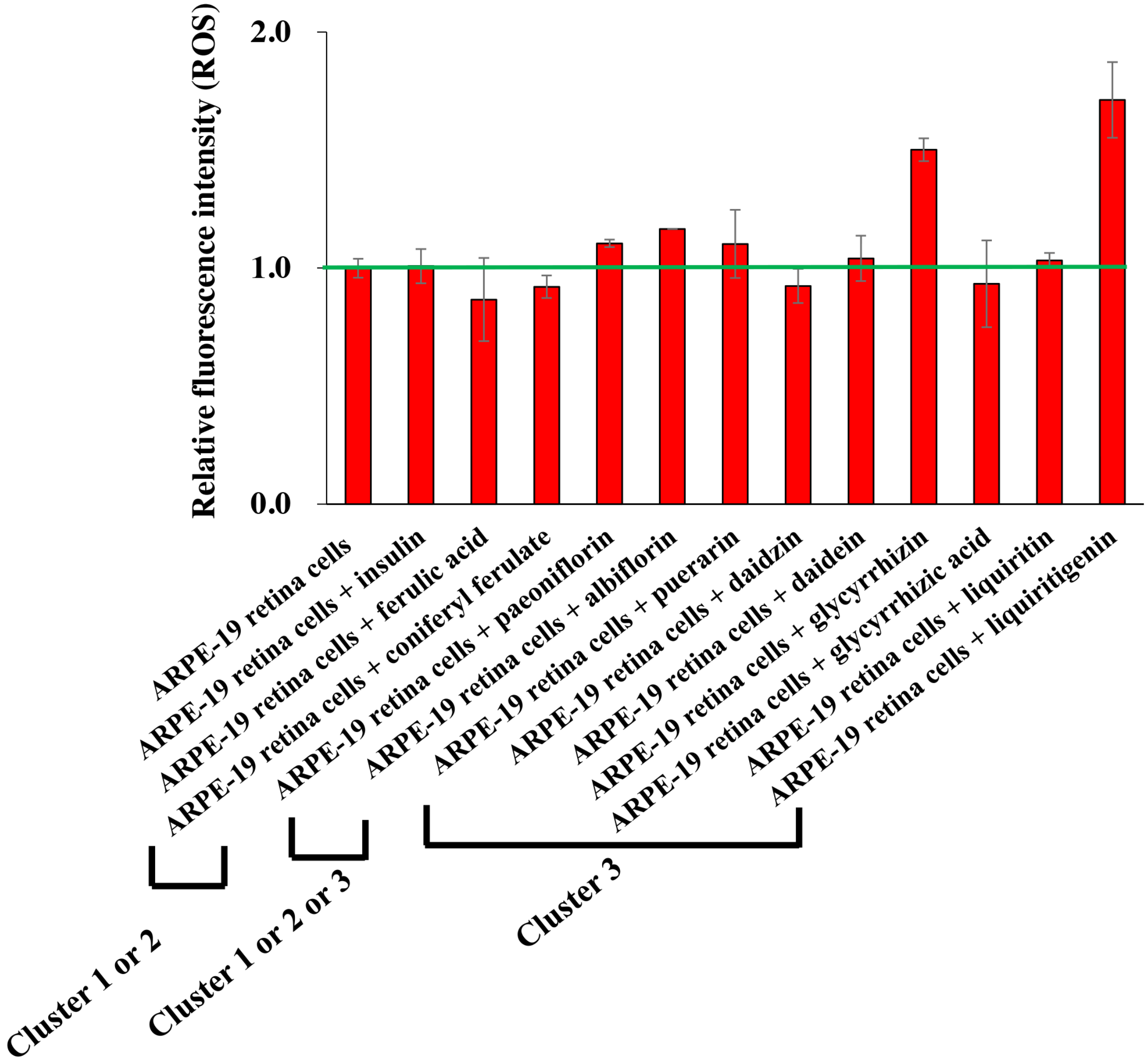

Supplementary Figure 6: Effects of natural compounds from clusters on intracellular ROS level in ARPE-19 retina cells. ARPE-19 retina cells were pretreated with or without insulin and natural compounds, respectively for 18 h. The treated cells were then stained with 5 µM DCFH-DA for 30 min. DCF fluorescence intensities were then measured by flow cytometry with an excitation wavelength of 488 nm and an emission wavelength of 535 nm, respectively (BD FACSCanto™ flow cytometry system, BD Biosciences, San Jose, CA). Relative fluorescence intensity (ROS) is expressed as relative intensity of the treated cells when compared with the un-treated control. Each value represents the mean±SEM of three independent experiments.

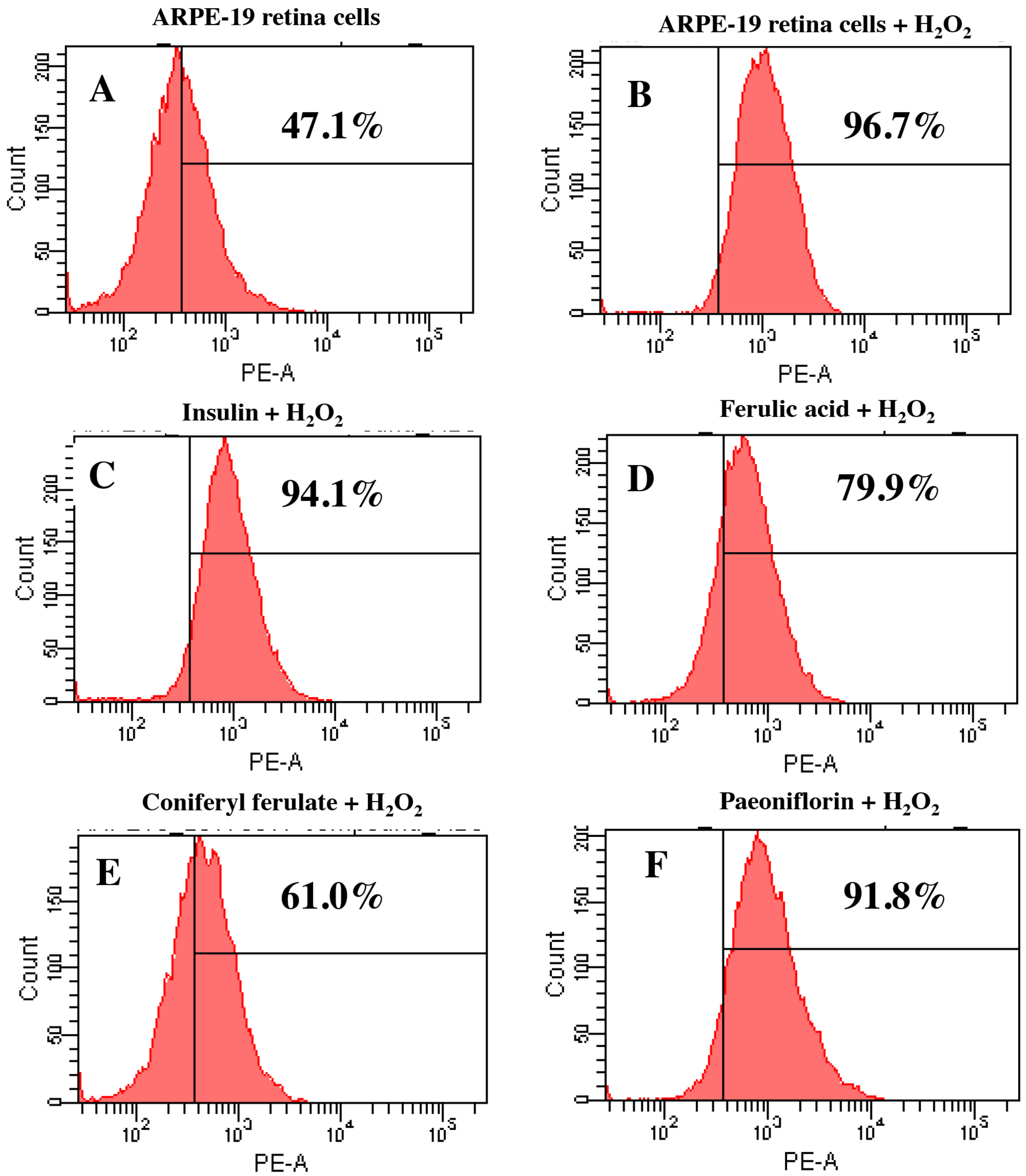
ARPE-19 retina cells
A
47.1%
Count
PE-A
ARPE-19 retina cells + H_2O_2
B
96.7%
Insulin + H_2O_2
C
94.1%
Ferulic acid + H_2O_2
D
79.9%
Coniferyl ferulate + H_2O_2
E
61.0%
Paeoniflorin + H_2O_2
F
91.8%

(Continued)

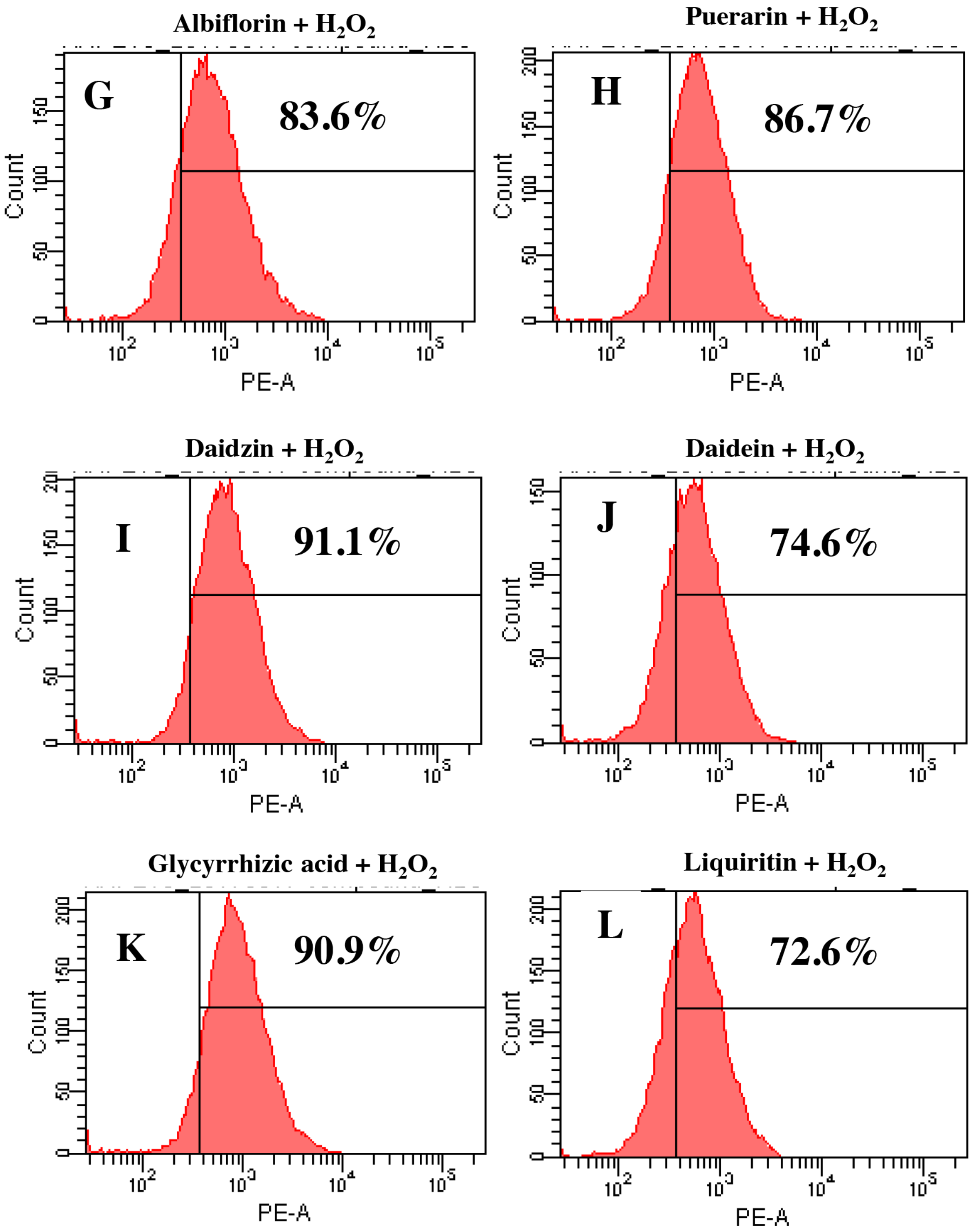
Albiflorin + H_2O_2
G
83.6%
Puerarin + H_2O_2
H
86.7%
Daidzin + H_2O_2
I
91.1%
Daidein + H_2O_2
J
74.6%
Glycyrrhizic acid + H_2O_2
K
90.9%
Liquiritin + H_2O_2
L
72.6%
Count
PE-A

(*Continued*)

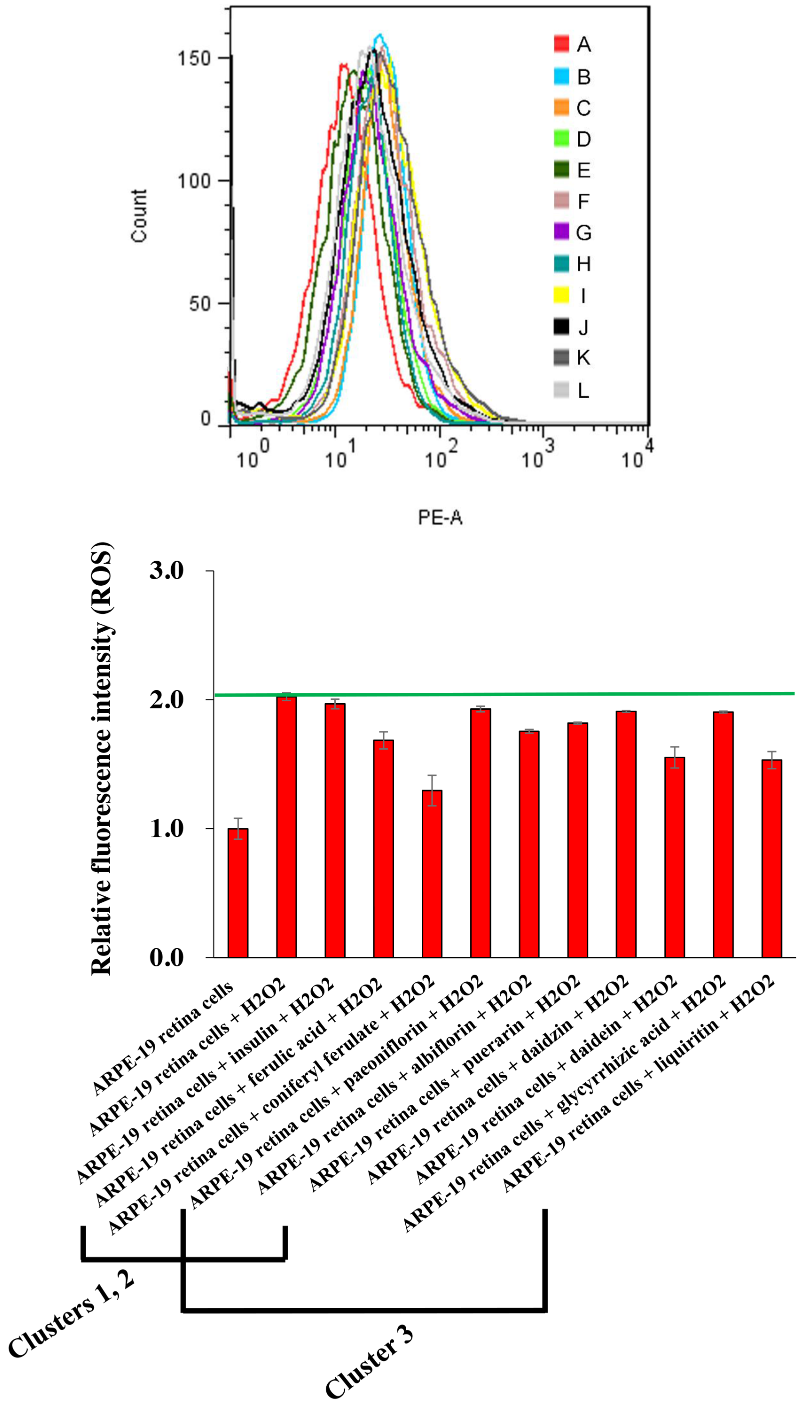

Supplementary Figure 7: Effects of natural compounds on hydrogen peroxide (H_2O_2)- induced ROS production in ARPE-19 retina cells. ARPE-19 retina cells were pretreated with or without insulin and natural compounds, respectively for 18 h. The treated cells were then stained with 5 µM DCFH-DA for 30 min and subsequently incubated with H_2O_2 (300 µM) for 5 min to induce the intracellular ROS generation. DCF fluorescence intensities were then measured by flow cytometry with an excitation wavelength of 488 nm and an emission wavelength of 535 nm, respectively (BD FACSCanto™ flow cytometry system, BD Biosciences, San Jose, CA). Relative fluorescence intensity (ROS) is expressed as relative intensity of the treated cells when compared with the un-treated control. Each value represents the mean±SEM of three independent experiments.
